# Theoretical Prediction of a Bi-Doped β-Antimonene Monolayer as a Highly Efficient Photocatalyst for Oxygen Reduction and Overall Water Splitting

**DOI:** 10.1021/acsami.1c18191

**Published:** 2021-11-16

**Authors:** Deobrat Singh, Rajeev Ahuja

**Affiliations:** † Condensed Matter Theory Group, Materials Theory Division, Department of Physics and Astronomy, Uppsala University, P.O. Box 516, Uppsala 75120, Sweden; ‡ Department of Physics, Indian Institute of Technology Ropar, Rupnagar 140001 Punjab, India

**Keywords:** 2D antimonene monolayer, overall water splitting, oxygen reduction, electronic properties, optical excitation, doping effect

## Abstract

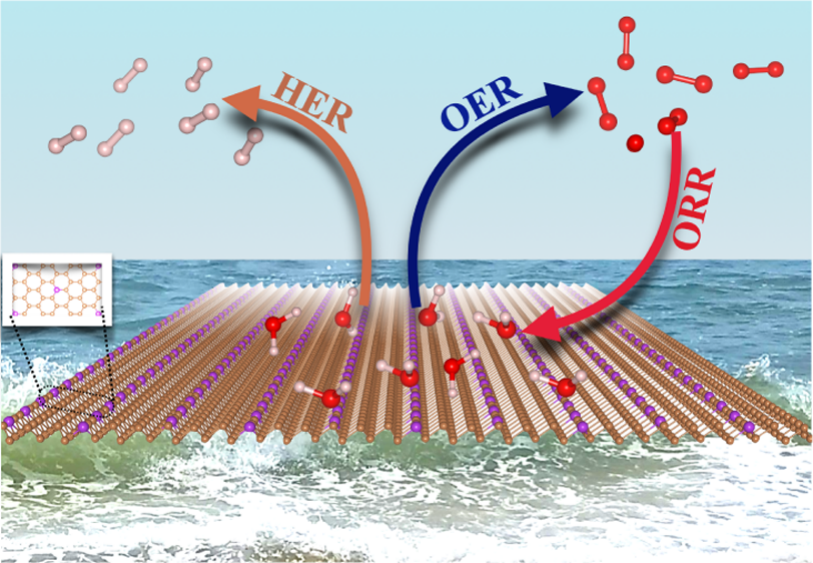

The photo-/electrocatalysts with high activities for the hydrogen evolution reaction (HER), oxygen evolution reaction (OER), and the oxygen reduction reaction (ORR) are of significance for the advancement of photo-/electrochemical energy systems such as solar energy to resolve the global energy crisis, reversible water electrolyzers, metal–air batteries, and fuel cells. In the present work, we have systematically investigated the photochemical performance of the 2D β-antimonene (β-Sb) monolayer. From density functional theory investigations, β-Sb with single-atom doping possesses a trifunctional photocatalyst with high energetics and thermal stabilities. In particular, it is predicted that the performance of the HER activity of β-Sb will be superior to most of the 2D materials. Specifically, β-Sb with single atom replacement has even superior that the reference catalysts IrO_2_(110) and Pt(111) with relatively low overpotential values for ORR and OER mechanisms. The superior catalytic performance of β-Sb has been described by its electronic structures, charge transfer mechanism, and suitable valence and conduction band edge positions versus normal hydrogen electrode. Meanwhile, the low overpotential of multifunctional photocatalysts of the Bi@β-Sb monolayer makes them show a remarkable performance in overall water splitting (0.06 V for HER, 0.25 V for OER, and 0.31 V for ORR). In general, the Bi@β-Sb monolayer may be an excellent trifunctional catalyst that exhibits high activity toward all electrode reactions of hydrogen and oxygen.

## Introduction

The rapid development of modern society needs cost-effective and high-performance photo-/electrocatalysts, which play critical roles in the storage and conversion of renewable energy, for example, hydrogen production from water electrolysis, rechargeable metal–air batteries, and fuel cells, including hydrogen evolution reaction (HER), oxygen evolution reaction (OER), and oxygen reduction reaction (ORR) mechanisms.^1−5^ So far, the advanced catalysts are still dominated by expensive noble metal or their oxides such as Pt for HER and ORR and RuO_2_, IrO_2_, and so forth for OER.^6,7^ In particular, the multistep proton and electron transfer process of oxygen electrode reactions such as OER/ORR with scale relationships is usually kinetically slow and proceeds with a high overpotential, which severely hamper their commercial applications.^8^ Thus, it is of great consequence and necessity to explore alternative nonprecious catalysts that are more efficient and durable.

Until now, the 2D layered materials have been considered as a hot research topic after the successful exfoliation of graphene.^9^ Recently, researchers have widely discussed several metal-free catalysts and bifunctional catalysts for the ORR/OER mechanism, including many types of layered 2D materials.^10−12^ The graphene-based layered materials, for example, graphene with N-doped and N-containing graphene co-doped with a second heteroatom (i.e., B, P, S, Fe, Co, Ni, etc.), have been shown to catalyze bifunctional for ORR and OER with high catalytic activity.^13^ Apart from this, various transition metal nitrides, sulfides, and phosphides have been successfully synthesized and extensively used in photocatalytic water decomposition.^14^ Most of them have shown immensely high carrier mobility and superior catalytic activity. For example, TMDs, MXenes, GeSe, GeS, and so forth have also been recognized as potential efficient catalysts.^3,15^ Also, it has recently been assumed that MoS_2_, a species of TMDs, is a distinguished performance catalyst for HER.^16^ However, MoS_2_ has a direct band gap of ∼2–3 eV in the single layer case.^17^ This deficiency limits its absorption of visible light, which in turn hampers its future applications.^18^ Subsequently, another layered material black phosphorous (BP) has an adjustable band gap with the layer thickness and the nature of the direct band gap remaining unchanged. BP also has high carrier mobility, and its instability in the air makes it difficult to be used in real applications. Inspired by the BP, elements of the same group of the V-group have gained enormous attention.

In the modern energy technologies, the bifunctional or trifunctional catalysts can be considered as a new concept for nanomaterial-based catalysts, and their use for water splitting through electrochemical reactions requires HER, OER, and ORR mechanisms.^19^ There has been a broad research focus on photo-/electrocatalytic nanomaterials based on precious and nonprecious metals or metal oxides as effective ORR, OER, and HER catalysts, whereas there have been very less studies on bifunctional catalysts for ORR/OER or HER/OER. Interestingly, high-performance trifunctional activity has rarely been reported for ORR/HER/OER mechanisms using carbon-based and non-carbon-based catalysts.^20−23^ The OER and ORR play an important role in oxygen electrodes via the charge and discharge mechanism of metal–air batteries, which demonstrate the overall performance of the device. In consequence, searching low-cost bifunctional/trifunctional catalytic nanomaterials that are efficient for ORR/OER or HER/OER or HER/OER/ORR mechanisms is a constant focus of research. Therefore, the development of such trifunctional catalysts would be suitable for different applications, for example, water splitting, metal–air batteries, fuel cells devices, and so forth.

Recently, the β-Sb monolayer has been successfully synthesized on different substrates by various experimental groups.^24,25^ The β-Sb monolayer has shown promising potential applications in the fields of optoelectronic devices,^26^ sensing devices,^27^ electrocatalysis,^28^ energy storage,^29^ and cancer therapy^30^ due to its unique chemical and physical properties as compared to conventional bulk materials, that is, high specific surface activity, interesting electronic property, moderate band gap, and superior carrier mobility.^31^ Moreover, metal atom-decorated β-Sb was theoretically investigated for ORR electrocatalysts.^32^ However, there has been no attempts on the implementation of the emerging 2D β-Sb monolayer structure in multifunctional photocatalysts.

Especially, the high strength of multifunctional catalysts lies in reducing the cost of the product due to reduced use of the equipment and fewer preparation procedures compared to the separate single-function catalysts.^33^ In the HER and OER mechanisms for overall water splitting, bifunctional catalysts always displayed better performance than two separate single-function catalysts because the optimum working conditions for two single-function catalysts are generally not the same.^33^ Therefore, it is more interesting to design and develop bifunctional or trifunctional catalysts. In the present work, we have investigated the emerging 2D β-Sb monolayer as a photocatalyst for HER, OER, and ORR using density functional theory calculations. We have also investigated the excitonic binding energy, absorption spectrum, and band edge position. The higher excitonic binding energy ≈0.60 eV displayed the suppressed fast recombination of photo-excited electrons and holes, which is very beneficial for photocatalytic activity. The β-Sb absorbs most of the light in the visible region. We have considered five different systems: pristine β-Sb, As@β-Sb, Bi@β-Sb, Sn@β-Sb, and Te@β-Sb for HER, OER, and ORR, in which the Bi@β-Sb monolayer system is the better candidate for multifunctional photocatalysts (HER/OER/ORR).

## Computational Methods

All the calculations have been performed using first-principles calculations as implemented in VASP software.^34^ The generalized gradient approximation in the form of Perdew–Burke–Ernzerhof functional (GGA-PBE) has been used for an exchange–correlation interaction.^35^ The van der Waals interactions with density functional theory (DFT)-D3 were considered by Grimme et al.^36^ For the plane-wave basis set, we have used an energy cutoff of 500 eV and (28 × 28 × 1) *k*-meshes for (1 × 1 × 1) unit cell of the β-Sb monolayer for Brillouin zone integration within the Monkhorst–Pack scheme.^37^ To describe the ion–electron interaction, we have used the projected augmented wave potential.^38^ Furthermore, we have used the (3 × 3 × 1) supercell with (6 × 6 × 1) *k*-meshes for the photocatalytic mechanism of the β-Sb monolayer. In addition, a vacuum of 20 Å has been used in the transverse directions to prevent the physical interactions between the consecutive layers. The convergence criteria for Hellmann–Feynman force fell below 2 × 10^–3^ eV/Å during the structural optimizations. The energy convergence criterion has been set as 10^–6^ eV for the electronic wave function.

It was seen that the standard DFT gives the underestimated band gaps; therefore, we have used the hybrid HSE06 functional for the accurate band gap with a screening parameter of 0.2 Å^–1^ and mixing parameter (α) of 25%.^39^ We have also utilized the hybrid functional B3LYP to determine accurate electronic and other properties. The optical properties of the β-Sb system were investigated using the BSE approach in addition to *G*_0_*W*_0_ calculation of a single shot,^40^ which was performed instead of the standard DFT calculations. The *G*_0_*W*_0_ plus BSE approach took into account the correlation effects of electron–electron (e–e) and electron–hole (e–h). We have investigated the phonon dispersion spectra using PHONOPY code^41^ with (3 × 3 × 1) supercell sheet to check dynamical stability. Ab initio molecular dynamics (AIMD) calculations were used for thermodynamical stability. Newton’s equation of motion is integrated using Verlet’s algorithm with time steps of 2 fs, and a nose thermostat is used for AIMD simulations. The stability of β-Sb and defected monolayer is determined at a high temperature (1000 K) using a canonical ensemble (*NVT,* i.e., fixed particle number, volume, and temperature) from AIMD simulation for 5 ps. AIMD calculations help to determine whether the change in structure is reversible or not, and it also provides information about the thermal stability of the used host material. Lobster software^42^ has been used to calculate the crystal orbital Hamiltonian population (COHP) for chemical bonding information. The climbing-image NEB approach has been used to investigate the energy barrier of intermediates and diffusion pathway for hydrogen and oxygen electrode reactions.

## Results and Discussion

### Structural Stability and Electronic Properties of β-Sb

**Figure 1:**
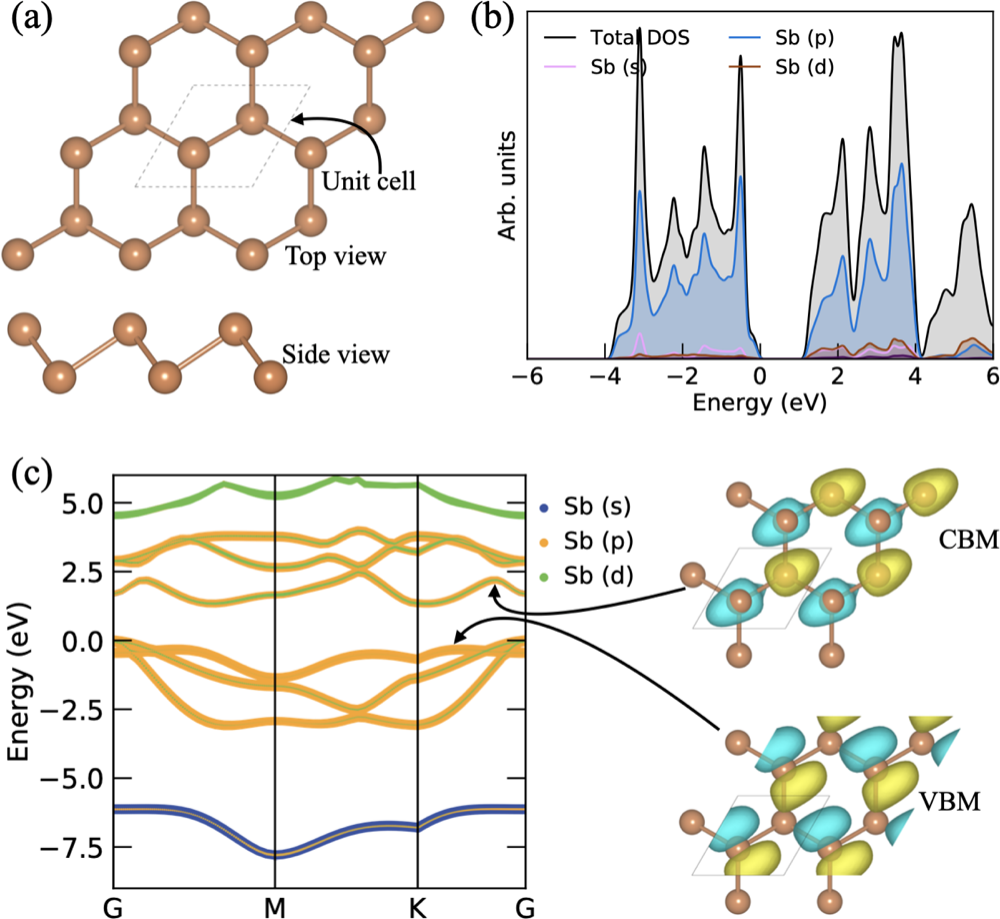
(a) Top and side views of the lowest energy configuration of pristine β-Sb, (b) projected DOS, and (c) corresponding decomposed electronic band structures with a wave function of VBM and CBM.

The crystal structure of β-Sb with top and side views is presented in Figure 1a. The unit cell of the β-Sb monolayer containing two atoms and the corresponding bond length and bond angle in Sb–Sb are found to be 2.88 Å and 90.1°. The lattice parameter has *a* = *b* = 4.07 Å, and these structural parameters are well consistent with the previously reported work.^26,31^ We have checked that the β-Sb monolayer has been energetically, dynamically, and thermally stable, which is confirmed by cohesive energy, phonon dispersion spectra, and AIMD calculations. The calculated cohesive energy of pristine β-Sb is −4.011 eV/atom, which is well consistent with the previously reported work.^31^ The cohesive energy values of β-Sb are relatively larger than most of the 2D monolayer systems. Additionally, the phonon band structure of β-Sb is presented in Figure S1a (see in Supporting Information). The vibrations of each mode have a positive frequency, which displayed the stability of the free-standing β-Sb monolayer. The maximum vibrational frequency is found to be 201.64 cm^–1^. Furthermore, we have investigated the AIMD to analyze the thermal stability of structures and to realize the possibility of experimental synthesis (see Figures S1–S3 in Supporting Information). To control the range of temperature to be around 1000 K for 5 ps with a time step of 2 fs has been investigated in the Nose algorithm. Figure S1b shows that the variation of total energy remains nearly constant with an increase in time, and Figure S3a (see in Supporting Information) displays the wild variation of temperature with an increase in time. It was seen that there is no breaking of the bond and no structural distortion that led to a thermodynamic stability of the structure, which confirmed the thermal stability.

To see the electronic behavior, we have calculated the electronic DOS and the corresponding band structures (see Figure 1b,c). From the projected PDOS, we can see that the main electronic contribution of Sb orbitals is near the Fermi level in the conduction and valence band. The p-states of Sb are more dominating near the Fermi level in both VBM (i.e., valence band maximum) and CBM (i.e., conduction band minimum), and small contributions come from its s-states. The VBM and CBM are separated from each other, which means that the β-Sb monolayer displayed semiconducting behavior. The β-sb monolayer has the band gap of 1.35 eV with the PBE functional, 1.99 with the HSE06 functional, 2.385 eV with the B3LYP functional, and 2.51 eV using the GW approach. Further, the decomposed orbital band structure shows that the p-states have a significant contribution in both VBM and CBM. Also, the s-states appear in the conduction band around 5 eV, and its d-states also appear in deep energy level around −7.5 eV in the valence band. Additionally, Figure 1c shows the wave function profile for VBM and CBM. From the electronic band structure, we can see that the initial interband transitions occur between bonding and antibonding p-orbital. We have computed the decomposed electronic band structure of split p-orbital (p_*x*_, p_*y*_ and p_*z*_) (see in Supporting Information). The possible interband transitions is mainly originated for the low photon energy region (up to 6 eV) from bonding states to anti-bonding states, that is, π → π*, π → σ*, and and σ → π*. These interband transitions (optical properties) will be discussed in the section below.

### Structural and Electronic Properties of Single-Atom Replacement in β-Sb

**Figure 2:**
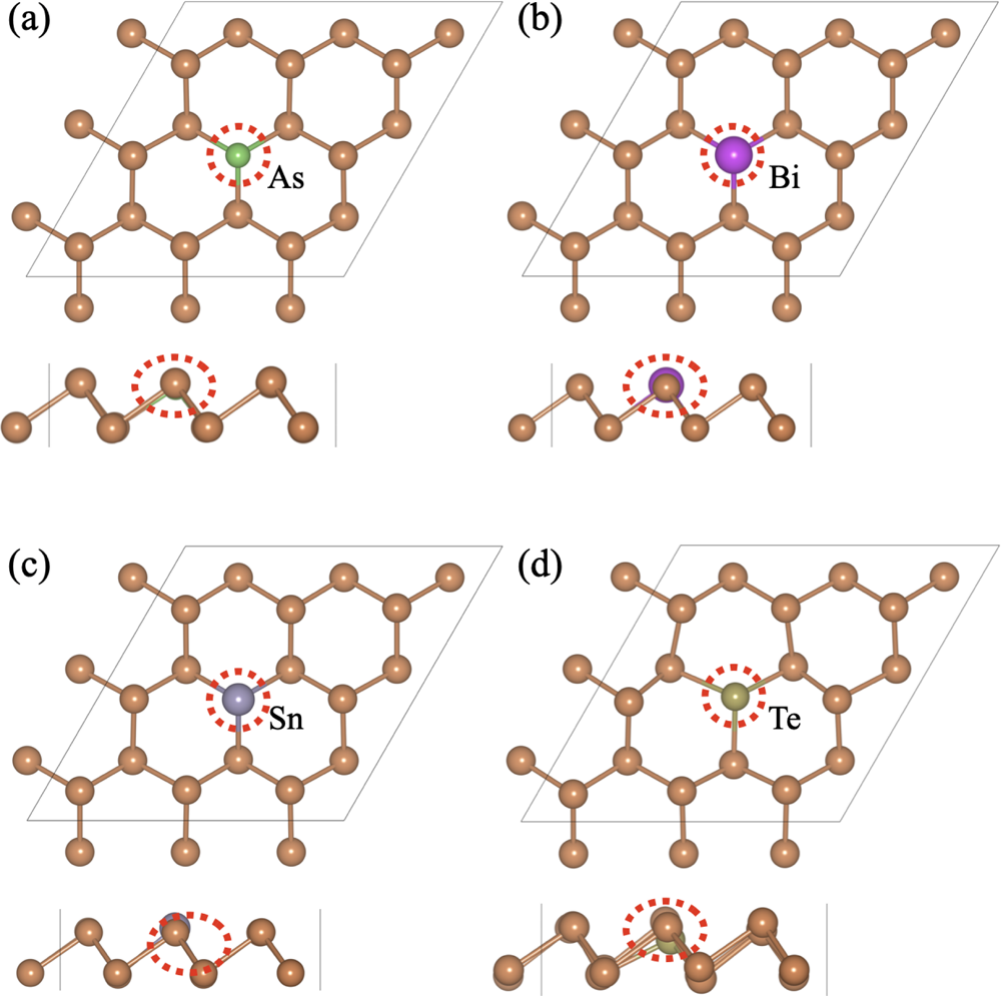
Lowest energy configurations of single-atom replacement from the β-Sb monolayer. Optimized structure of (a) As@β-Sb, (b) Bi@β-Sb, (c) Sn@β-Sb, and (d) Te@β-Sb systems.

Figure 2 shows the fully optimized structures with top and side views of single-atom replacement from the β-Sb monolayer by As, Bi, Sn, and Te. The replacement atom is presented by a circle with red color. When a single Sb is replaced with As, then the angle between Sb–Sb–Sb (90.2°) shows a negligible effect, while the angle between Sb–As–Sb is slightly increased and found to be 92.4°. The bond length (2.875 Å) between Sb–Sb is the same as the pristine β-Sb monolayer, while the Sb–As bond length is slightly decreased and found to be 2.71 Å. Similarly, when the Sb atom is replaced with Bi, Sn, and Te atoms, the Sb–Sb bond length was found to be 2.876, 2.88, and 2.878 Å, while Sb–Bi, Sb–Sn, and Sb–Te lengths are 2.947, 3.0, and 3.024 Å and the corresponding angles are 90.5–88.4°, 91.0–84.0°, and 91.7–98.5°, respectively. There is no structural distortion due to the very few variations in the bond length of Sb–Sb and bond angles. Further, to check the stability of a single-atom replacement of the β-Sb monolayer, we have investigated the cohesive energy for these materials. The calculated cohesive energies of As@β-Sb, Bi@β-Sb, Sn@β-Sb, and Te@β-Sb systems are −4.13, −4.09, −4.05, and −4.02 eV/atom, respectively, which are very close to that of the pristine β-Sb monolayer. It means that the single-atom replacement β-Sb systems are energetically stable. Further, verification was done by AIMD simulations (see Figures S2 and S3 in Supporting Information) at high temperatures (1000 K), which indicated the stable structure with no structural distortion and no breaking of bonds. It means that the single-atom replacement β-Sb monolayer confirmed the thermal stability.

**Figure 3:**
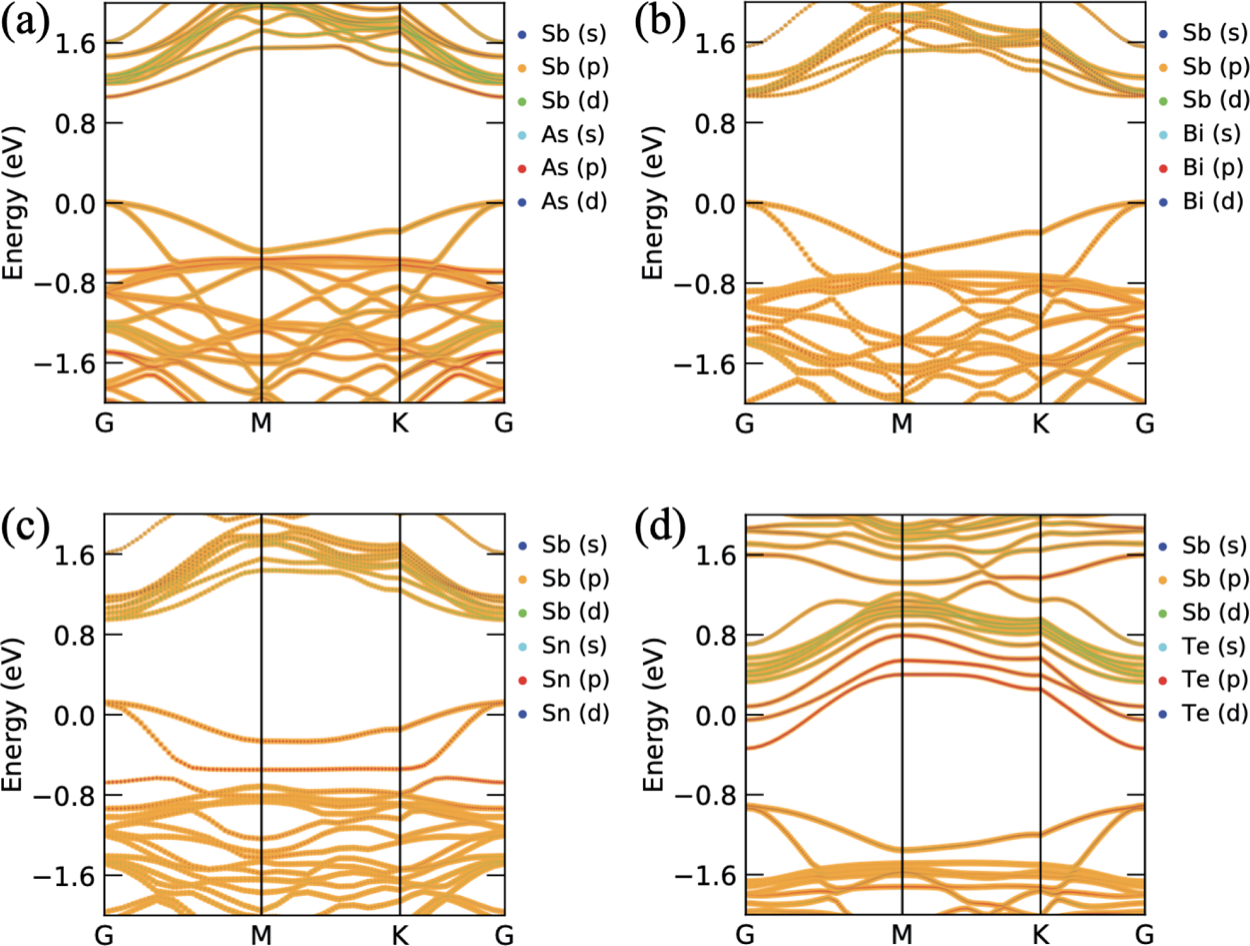
Orbital contributed band structures of (a) As@β-Sb, (b) Bi@β-Sb, (c) Sn@β-Sb, and (d) Te@β-Sb systems.

Due to the variation in bond length and bond angle, the electronic properties significantly changed, and it is shown in Figure 3. As@β-Sb and Bi@β-Sb systems show the semiconducting behavior with the direct band gaps of 1.062 and 1.064 eV at G-point, respectively. The electronic band gap significantly reduced from 1.35 to ≈1 eV. The VBM is made by Sb orbitals, while the CBM is originated by the As/Bi atom. It means that the replacement of the Sb atom with As/Bi reduced the band gap of the pristine Sb monolayer. Sn and Te atom replacement in place of Sb leads to change in behavior from semiconducting to metallic because Sb orbitals are strongly hybridized with Sn/Te orbitals at the Fermi level. In both Sn/Te cases, two electronic band lines cross the Fermi level. It is evident from Figure S4 (see in Supporting Information) that higher charge density near to the replaced atom (see Figure 2), followed by their charge, accumulates around the As, Bi, Sn, and Te atoms. Also, it was seen that the overall charges are redistributed over the Sb surface. From the Bader charge analysis, the As atom gains 0.53 electrons from Sb atoms because As atoms have more electronegativity than the Sb atoms. That is why the tendency of electron capturing capacity of As is higher than Sb. Similar to As atoms, the Te atom also has higher electronegativity; therefore, in this case, Te gains 0.37 electrons from the three neighboring Sb atoms. Apart from this, Bi and Sn atoms have less electronegativity as compared to Sb atoms; that is why the Sb atom attracts some electrons from the Bi and Sn atoms. The Bi atom loses 0.061 electrons and the Sn atom loses 0.20 electrons.

Furthermore, we have carried out the crystal orbital Hamilton population (COHP) analysis to check the chemical bonding between the atoms (see Figure S5 in Supporting Information). COHP splits the energy of the band structure into different orbital pair interactions, which can be used to index the binding, nonbinding, and antibonding contributions to the band structure. The projected COPH analysis gives a quantitative estimate to see the bond strengths in the structures with the help of −pCOHP values. In Figure S5, bonding and antibonding are represented by positive (+*y*-axis) and negative (−*y*-axis) signs, respectively. We have calculated the −pCOHP for the different bond pairs Sb–Sb, Sb–As, Sb–Bi, Sb–Sn, and Sb–Te in the crystal structures of pristine β-Sb, As@β-Sb, Bi@β-Sb, Sn@β-Sb, and Te@β-Sb monolayers. In the case of the pristine β-Sb monolayer, we observed no significant antibonding contributions in the Sb–Sb bond below the Fermi level (see Figure S5a in Supporting Information). Also, the integrated COHP (ICOHP) value shows the negative value, which represents the strong covalent interactions, whereas its positive value shows the weaker bonding interactions. Here, the pristine β-Sb monolayer shows the negative −ICOHP value; it means that the Sb–Sb bond shows the covalent interaction analyzed by the COHP framework. Similar to the pristine β-Sb monolayer, the As@β-Sb and Bi@β-Sb monolayers display the covalent interaction because there is no significant antibonding contribution observed in Sb–Sb and Sb–As/Sb–Bi pairs (see Figure S5b,c in Supporting Information). Also, the ICOHP value shows negative values of these pairs, whereas Sn@β-Sb and Te@β-Sb monolayers represent the antibonding contributions at the Fermi level, indicating that the covalent interaction weakens in Sb–Sn and Sb–Te pairs (see Figure S5d,e in Supporting Information). Consequently, Sn@β-Sb and Te@β-Sb monolayers represent low stability, and thus they display poor catalytic performance.

### Hydrogen Evolution Reaction

Generally, Gibbs free energy of adsorption of intermediate hydrogen (Δ*G*_H*_) on the catalyst provides information to analyze the hydrogen evolution reaction (HER) activity performance. The values of Δ*G*_H*_ should be zero for an ideal catalyst for the HER mechanism.^43^ Initially, we have considered three different adsorption sites (just above the Sb atom, between the Sb–Sb bond, and between the hexagonal lattice arrangement of Sb atoms), in which we have taken the most favorable lowest energy configuration (see Figure S6a–e in Supporting Information). In the case of pristine β-Sb, the H atom is placed at the center of the hexagonal lattice arrangement after the full optimization, the H atom is shifted toward the Sb atom, and the H atom is bonded with two Sb atoms. The optimized structure of pristine β-Sb with H atoms slightly changed the bond length between Sb–Sb near the H atoms. The slightly deformed bond lengths are found to be 2.866 Å from 2.88 Å and Sb–H bond length is found to be 2.04 Å. Similarly, the H atom is adsorbed on the surface of the As@β-Sb, Bi@β-Sb, Sn@β-Sb, and Te@β-Sb systems, in which the bond lengths between As–H are 1.54, 2.43, 2.23, and 3.46 Å for As@β-Sb, Bi@β-Sb, Sn@β-Sb, and Te@β-Sb systems, respectively. In general, the criterion to judge whether a material has HER activity follows the classic rule |Δ*G*_H*_|, which must be less than 0.2 eV.^44^

**Figure 4:**
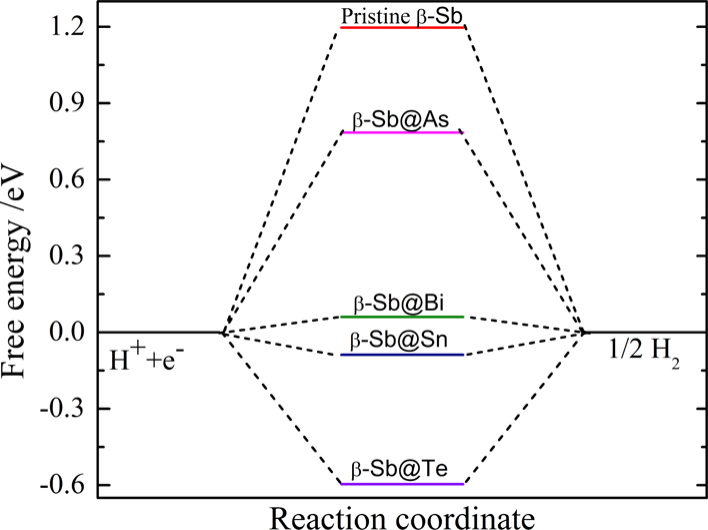
Free energy profile of HER on the pristine β-Sb monolayer and single Sb atom replaced by As, Bi, Sn, and Te atoms as represented by As@β-Sb, Bi@β-Sb, Sn@β-Sb, and Te@β-Sb, respectively, using the hybrid B3LYP functional.

The lowest free energies are found to be −0.09 and −0.077 eV using the GGA-PBE functional in the case of Te@β-Sb and Bi@β-Sb configurations. Due to the presence of very low free energy of Bi@β-Sb configuration, we have further calculated the free energy of Bi@β-Sb configuration using the hybrid B3LYP functional and found that it is 0.06 eV. For the comparison of other configurations, we have plotted the Gibbs free energy diagram, as shown in Figure 4. It was seen that the performance of HER activity of the defected β-Sb monolayer is superior to the MoS_2_ (2.08 eV), MoS_2_–Mo edge (−0.36 eV), PtS_2_ (0.86 eV), PdTe_2_ (0.74 eV), PtTe_2_ (0.54 eV), and PtSe_2_ (0.63 eV) systems.^45−48^ Additionally, the performance of HER activity of the β-Sb monolayer is equal to the novel metal Pt (−0.09 eV),^49^ slightly higher than the Pd monolayer (−0.04 eV)^50^ and slightly lower than Pd (−0.30 eV).^51^ It means that the single replacement in the β-Sb monolayer with different atoms displayed the superior candidates for HER performance. It was also seen that the HER activity is very sensitive to the hydrogen coverage.^1^ Therefore, we have investigated the Gibbs free energy with different hydrogen coverages from 1/8 to 8/8. To see the superior HER activity of β-Sb with single-atom doped as well as pristine configuration, we have considered it with different hydrogen coverages to calculate Gibbs free energy. The Gibbs free energy at different hydrogen coverages 2/8, 3/8, 4/8, and 8/8 are found to be 0.97/0.41/0.14/0.30, 0.97/0.60/0.11/0.29, 0.77/0.58/0.36/0.35, and 0.62/0.48/0.42/0.42 for pristine β-Sb, As@β-Sb, Sn@β-Sb, and Te@β-Sb, respectively. The values of |Δ*G*_H*_| for Bi@β-Sb at lower hydrogen coverage 2/8 and 3/8 are 0.236 and 0.53 eV, respectively. Moreover, at higher hydrogen coverage 4/8 and 8/8, the values of |Δ*G*_H*_| are found to be 0.108 and 0.11 eV, respectively. It means that the Bi@β-Sb monolayer exhibits excellent HER performance under higher H coverage 4/8 and above.

Furthermore, we have analyzed the charge transfer between surface and H atom using Bader analysis.^52^ When H atoms are adsorbed on the pristine β-Sb surface, then Sb atoms transfer 0.37 e to H atoms. Similarly, H atoms gain 1.0, 0.95, 0.90, and 0.99 e from As@β-Sb, Bi@β-Sb, Sn@β-Sb, and Te@β-Sb surfaces, respectively. Moreover, we have plotted the charge density difference profile of the most active configuration for HER activity using the relation Δρ = ρ_β-Sb@X+H_ – ρ_β-Sb@X_ – ρ_H_, which show the charge redistribution with the adsorption of H atoms on the surface of the β-Sb monolayer. Here, X = As, Bi, Sn, and Te. Figure S7 shows the charge density difference profile of As@β-Sb, Bi@β-Sb, Sn@β-Sb, and Te@β-Sb systems. The electron accumulation and charge depletion are represented by yellow and blue color, respectively (see Figure S7a–d in Supporting Information).

**Figure 5:**
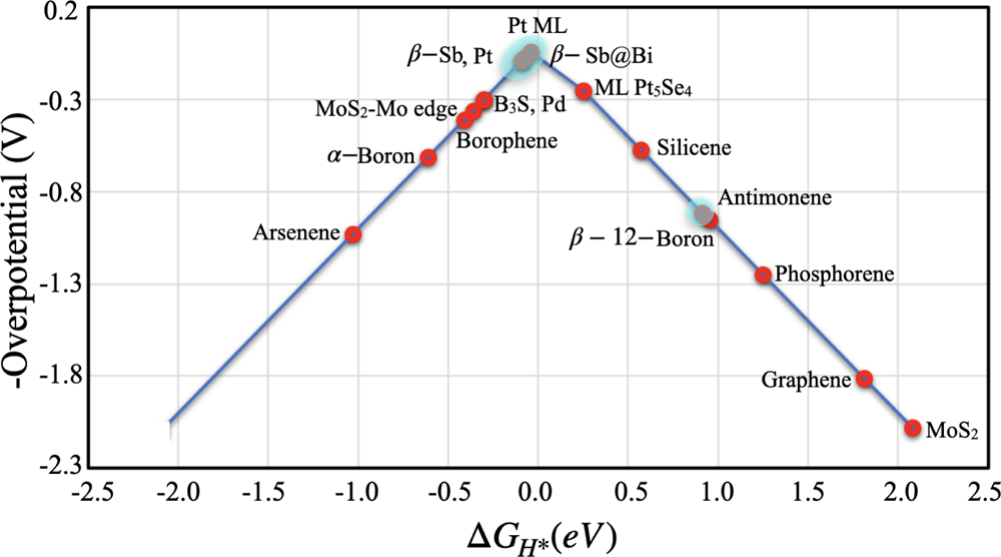
HER volcano curve of β-Sb (light blue circle) compared to the previously reported list of 2D layered materials^51,53−58^ including the most commonly used Pt.^49^ The values of overpotential in the present work are highlighted in light blue.

A volcanic curve is depicted in Figure 5 to compare the HER performance of the β-Sb monolayer with other 2D layered materials and well-studied Pt catalysts. The closest values at the top of the volcanic curve display the higher catalytic activity. It can be clearly seen that the HER catalytic activity of the 2D β-Sb monolayer is much higher than the corresponding other 2D monolayered materials. This means that the optimization of the β-Sb monolayer with single-atom replacement enhances the performance of the catalytic activity of HER. Moreover, the catalytic activity of the Bi@β-Sb monolayer is relatively better than the Pt catalyst, which means that the Bi@β-Sb monolayer could be a promising candidate for HER activity.

**Figure 6:**
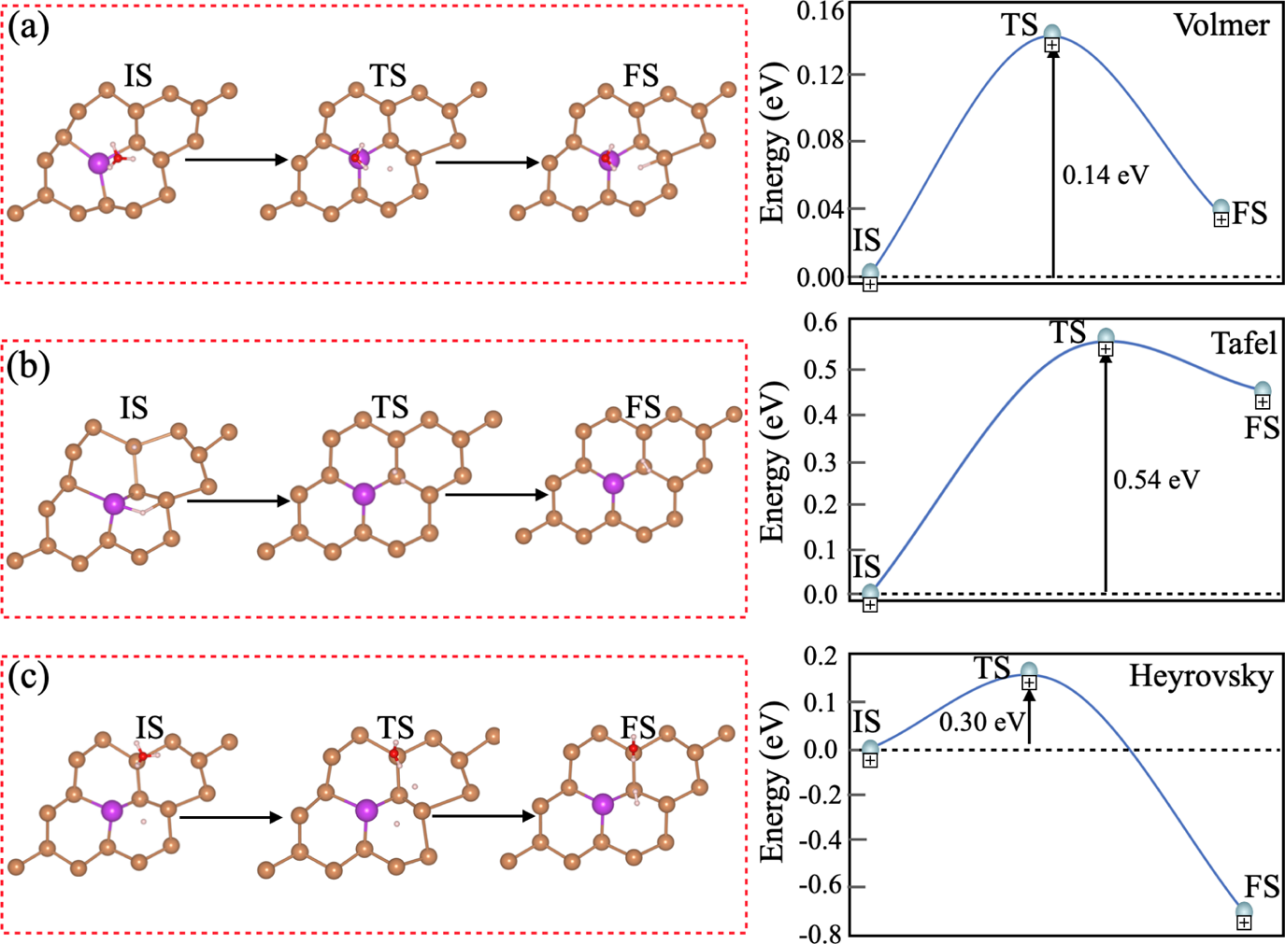
HER reaction pathways of Volmer–Tafel and Volmer–Heyrovsky reactions. The activation barriers for (a) Volmer, (b) Tafel, and (c) Heyrovsky reactions.

Moreover, Heyrovsky and Tafel reactions for the HER mechanism in an environment of pH = 0 (acidic medium) are more thoroughly investigated to better understand the reaction mechanism in the production of H_2_ on the surface of the Bi@β-Sb monolayer. Figure 6 displays HER, and the adsorption of hydrogen (Volmer reaction) and Heyrovsky and Tafel reactions are presented by
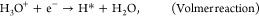
1
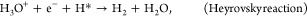
2
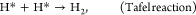
3

In the case of Volmer reaction, H* is adsorbed when the surface of the Bi@β-Sb monolayer interacts with the H_3_O^+^ cluster. After that, again the H_3_O^+^ group interacts with the already adsorbed H* with the surface of the Bi@β-Sb monolayer, which produced H_2_ in the case of Heyrovsky reaction. The activation barrier is examined by breaking of a proton of the H_3_O^+^ group. The activation barrier for the Heyrovsky pathway is found to be 0.30 eV (Figure 6c). In the case of the Tafel reaction, the activation barrier is 0.54 eV, as presented in Figure 6b. The activation barriers of the hydrogen evolution on the surface of the Bi@β-Sb monolayer follows the Volmer–Heyrovsky (0.30 eV) rather than the Volmer–Tafel (0.54 eV) pathways. Under the low overpotential and rapid kinetics, b-PtM is an attractive candidate for HER photocatalysts.

### Oxygen Evolution Reaction/Oxygen Reduction Reaction

**Figure 7:**
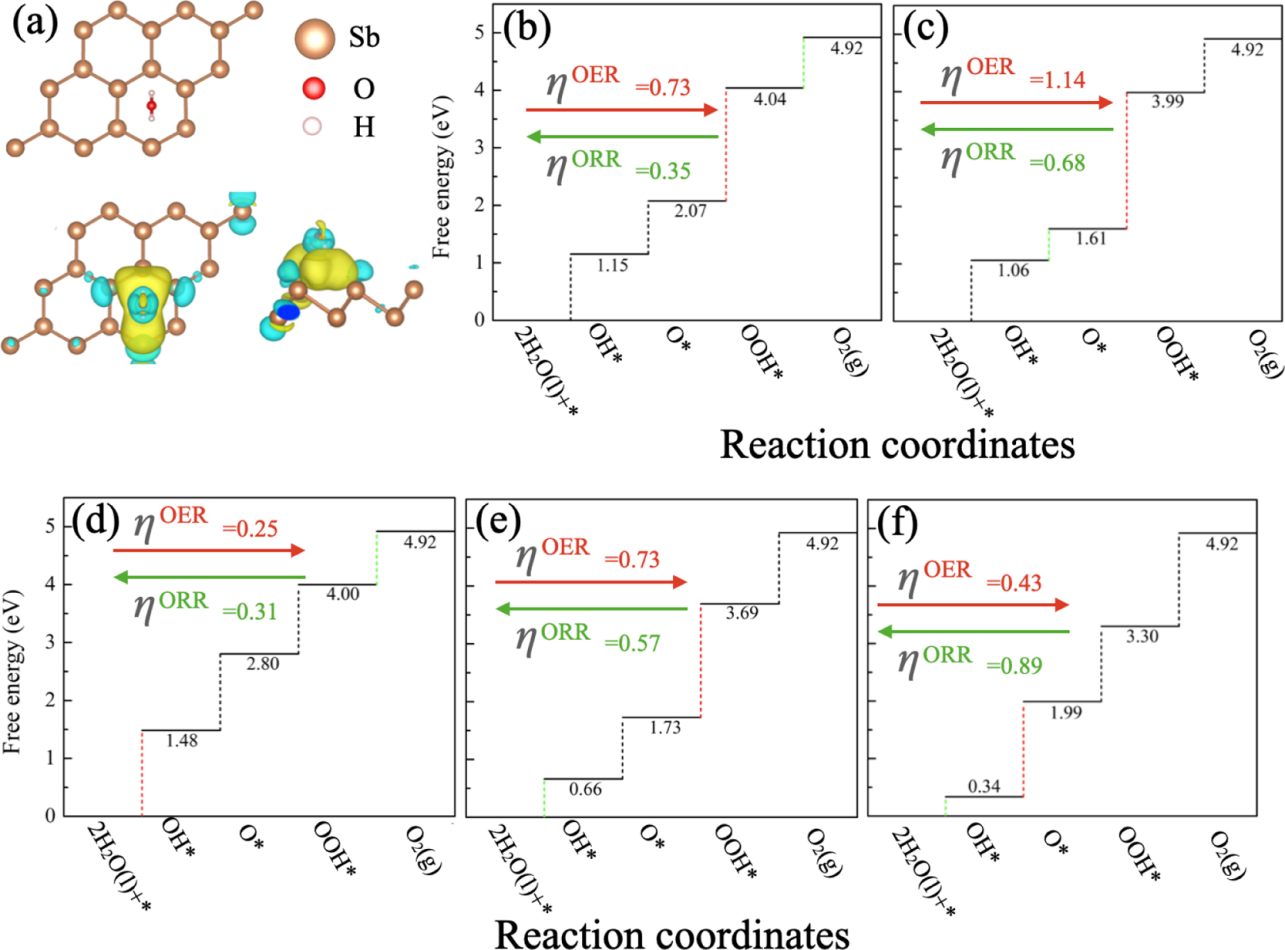
(a) Lowest energy configuration of H_2_O adsorption and the corresponding charge density profile for H_2_O adsorbed on β-Sb monolayer. The charge accumulation and depletion regions are represented by yellow and blue color, respectively, and 0.13 × 10^–3^ e/Å^–3^ has been set as the isosurface value. The free energy profile of OER and ORR mechanisms on the β-Sb monolayer and the corresponding OER and ORR mechanisms is presented in (b) pristine β-Sb, (c) As@β-Sb, (d) Bi@β-Sb, (e) Sn@β-Sb, and (f) Te@β-Sb monolayer at the *U* = 0 V. The red and the green dotted lines/arrows represent the rate-determining step for OER and ORR, respectively.

Now, the performance of OER/ORR activity for the pristine β-Sb, As@β-Sb, Bi@β-Sb, Sn@β-Sb, and Te@β-Sb monolayer systems has been investigated. Initially, we checked the lowest energy configuration for the water (H_2_O) on the surface of the β-Sb monolayer (see Figure 7a). The adsorbed H_2_O molecules are slightly deformed, and deformed band lengths between O–H are 0.973 and 0.974 Å from 0.969 Å and the bond length between H–O–H is 103.6° from 104.0°. The adsorption energy of H_2_O molecules is about −0.19 eV, which is larger than that of MoS_2_ and it is the Janus MoSSe monolayer.^59^ Moreover, charge transport and charge density difference profile confirmed that the significant charge transfer occurs between the adsorbed species and β-Sb monolayer. It means that adsorbed H_2_O molecules can be activated substantially by the activation site of β-Sb catalysts. Also, relatively larger interaction of H_2_O molecules with the β-Sb monolayer may confirm better photocatalytic activity splitting water.

The reaction mechanism of OER is complicated in four elementary reaction pathways:^33^ (a) initially the H_2_O molecules dissociate into two parts H^+^ and HO* on the surface of the catalyst, (b) HO* further dissociates in two parts H^+^ and O*, (c) then O* reacts with the next H_2_O molecules which produce H^+^ and HOO*, and (d) finally the HOO* splits into two parts, H^+^ and O_2_ molecules, and then O_2_ is released from the surface of the catalyst. The reaction mechanism for OER is presented in Supporting Information. An electron and a cation H^+^ are always released simultaneously in each elementary step, as depicted in Figure S8 (see in Supporting Information). In the OER mechanism, at every reaction step, the release of a H^+^ cation and electron occurs simultaneously. The lowest energy configurations of all possible intermediates for OER (O*, HO*, and HOO*) on the pristine β-Sb surface and defected systems are depicted in Figure S9 (see in Supporting Information), and the corresponding free energies for every step that follows the uphill reactions are listed in Figure 7. The overpotential (η_OER_) values of OER can be achieved based on the Gibbs free energy values for each reaction step (see Supporting Information). The rate-determining step for OER is denoted by the dotted red line in Figure 7 at electrode potential of *U* = 0 V. We have considered five different systems for the OER mechanism, in which the overpotential values (η_OER_) for the pristine β-Sb monolayer is found to be 0.73 V (η_OER_ = 4.04–2.08–1.23 V) using the hybrid B3LYP functional and the transformation of O* to HOO* is the rate-determining step. It was seen that the calculated overpotential η_OER_ of the pristine β-Sb monolayer is slightly lower than the considerably studied catalyst, for example, Pt(111) surface (0.76 V),^60^ and slightly larger than the metal atom-decorated C_2_N monolayer (0.67 V)^61^ and metal-decorated boron monolayer (0.40–2.12 V).^33^ Furthermore, we have investigated the overpotential values for replacing one Sb atom with As, Bi (in the same group), and Sn and Te (different group left and right element in the periodic table), as depicted in Figure 7c–f. With the replacement of different group elements, overpotential values (η_OER_) are found to be 0.73 V for Sn@β-Sb and 0.43 V for Te@β-Sb systems using the hybrid B3LYP functional. Moreover, the overpotential values (η_OER_) are found to be 1.14 and 0.25 V in the case of the same group atom replacement for As@β-Sb and Bi@β-Sb, respectively, using the hybrid B3LYP functional. Only the Bi@β-Sb monolayer system exhibits better catalytic activity η_OER_ as compared to the previously reported values for RuO_2_ (0.42 V)^62^ and IrO_2_ (0.56 V),^62^ PtM with low Pt loading,^51^ and CoN_*X*_ (0.69–1.81 V),^63^ which suggest that the Bi@β-Sb monolayer can act as a superior catalyst candidate for the OER mechanism.

**Figure 8:**
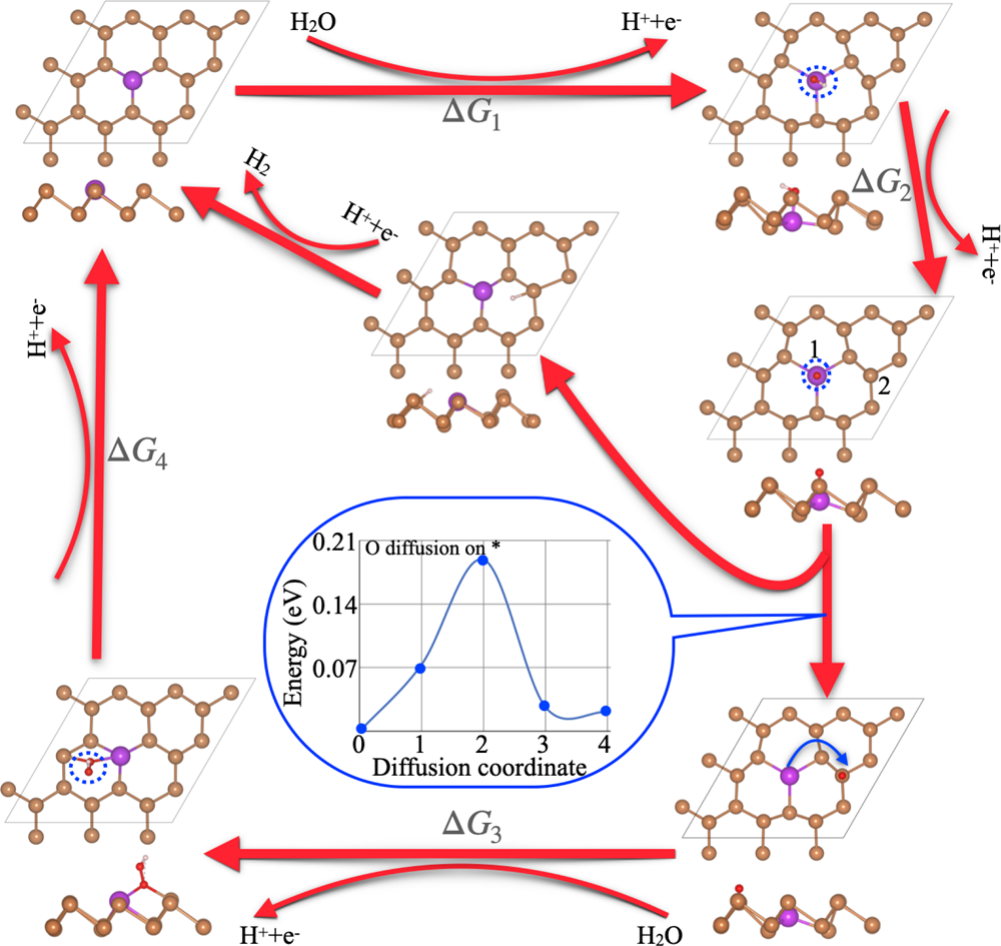
Reaction process of the whole OER mechanism and intermediates have optimized the lowest configuration for the dual-active site mechanisms. The inset displays the HER mechanism for the Bi@β-Sb system. The inset with blue color represents the activation energy barrier of O* from the catalytically active site 1 to site 2.

Figure 8 displays the overall OER reaction mechanism, and the corresponding OER profile shows the dual-active site mechanism of the intermediate step. It was seen that the Bi@β-Sb monolayer displayed the better OER activity therefore we have investigated the different active adsorption sites and we have studied the migration behavior of the intermediate state at different active sites (presented as site 1 and site 2 in Figure 8). The inset encircled in blue color shows that the diffusion energy barrier of O* is 0.19 eV from site 1 to site 2. The significantly small value of activation energy and site-2 shows most stable configuration, indicating that once O* is formed, it rapidly transfers from site 1 to site 2, which is the next elemental reaction to form HOO*. It strongly confirms the viability of the dual active site mechanism. Additionally, the dual active site reaction appears in the case of the OER process when O* is transferred from site 1 to site 2, and then vacant active site 1 can perform the HER mechanism by producing H^+^ from the OER process. It means that, site 1 also act as an active site for the HER mechanism. This further accelerates the kinetic rate for efficiency of the overall water splitting.

Now, we proceed to the ORR mechanism, which is the opposite reaction mechanism of OER (see Supporting Information).^64^ Using the mechanism of the four-elementary reaction, the overpotential for ORR (η_ORR_) can be calculated by eq S19 (see in Supporting Information), and it is calculated by the minimum step distance (rate-determining step) as presented by dotted green color in Figure 7. Each step of the ORR mechanism for each system displayed the downhill in the free energy diagram, specifying that each reaction proceeds spontaneously. The overpotential values for the pristine β-Sb monolayer, As@β-Sb, Bi@β-Sb, Sn@β-Sb, and Te@β-Sb systems, are 0.35, 0.68, 0.31, 0.57, and 0.89 V, respectively, obtained using the hybrid B3LYP functional. The overpotential values of the studied system pristine β-Sb monolayer and Bi@β-Sb are relatively lower than that of the well-defined catalyst Pt of 0.45 V,^64^ indicating that the pristine β-Sb monolayer and Bi@β-Sb system are excellent candidates for the ORR mechanism. From these results, we can say that Bi@β-Sb is an excellent multifunctional photocatalyst for HER/OER/ORR.

The Bi@β-Sb monolayer is an efficient trifunctional photocatalyst for HER, OER, and ORR mechanisms. Therefore, we have analyzed the bonding information via COHP analysis in Sb–Sb and Sb–Bi pairs when H, O, OH, and OOH are adsorbed on the surface of the Bi@β-Sb monolayer. Figure S10 (see in Supporting Information) shows the COHP analysis to check the chemical bonding information. Here, the stabilized covalent interactions can be seen in Sb–Sb atoms when the H atom is adsorbed on the Bi@β-Sb monolayer. Massive bonding states occur below the Fermi level, while the antibonding states appear above the Fermi level, and also the -ICOHP value is negative, which means that the pairs Sb–Sb and Sb–Bi atoms form strong covalent interactions. Similarly, when O,OH, and OOH species are adsorbed on the Bi@β-Sb monolayer, it displayed the covalent interaction between the pairs in the host compounds. It means that with the adsorption of O, OH, and OOH species on the Bi@β-Sb monolayer, the covalent bond remains stronger, which enhances the catalytic performance of the materials.

### Optical Excitation and Photocatalytic Behavior

Figure S11 (see in Supporting Information) shows Im(ε) and the corresponding optical transitions strength. The e-h interaction included in GW plus BSE for incident light polarized along lattice vector *a* = *b* direction and optical absorption are dominated by excitonic states in one particular direction. The first absorption peak appears at 1.909 eV corresponding to the excitation energy. This peak mainly appears via a state of a bound exciton, and it originates with the transition from valence and conduction band (i.e., π → π*) in the Γ point of high symmetry (see Figure S12 in Supporting Information). In addition, the excitonic binding energy *E*_B_ (=*E*_g_^GW^ – *E*_g_^optical^) of 0.604 eV, which is well consistent with previous literature.^65^ The larger *E*_B_ can strongly confine the holes and electrons. Consequently, it suppresses the fast recombination of photogenerated holes and electrons. It suggests that the β-Sb monolayer is the superior candidate for photocatalytic activity and optoelectronic devices. Apart from this, the other two excitonic peaks are found at 2.54 and 3.35 eV, which lie very near to the visible region. It can be seen that the most of sunlight absorbed the visible region and starting of the ultraviolet (UV) region mainly up to 4 eV. Furthermore, we have calculated the light absorption spectrum of the β-Sb monolayer using the GW plus BSE method superimposed with the incident AM1.5G solar flux (see Figure S13 in Supporting Information). From Figure S13, it can be seen that most of the light was covered by the solar spectrum (including the visible light and near UV) for the β-Sb monolayer, which means that this material is very useful to make photovoltaic devices.

The photocatalytic water splitting is an efficient approach for storage and conversion of solar energy to meet energy demand.^66^ Due to the maximal specific surface, the 2D monolayer materials are promising materials for photocatalysis^67^ and can accelerate the migration of holes and electrons to the reaction interfaces.^68^ Generally, the water-splitting photocatalyst must satisfy two requirements: moderate band gap (i.e. >1.23 eV) and suitable alignment of band (band edge positions covering the redox potential of the water).^69^ The standard redox potential versus normal hydrogen electrode for pH = 0 are positioned at 0 eV for H^+^/H_2_ reduction potential and 1.23 eV for oxidation potential of O_2_/H_2_O of water. From the Nernst equation, the redox potentials of water will increase with pH and it can be expressed as: *E*_H^+^/H_2__ = (0.0 + pH × 0.059) eV and *E*_O_2_/H2O_ = (1.23 + pH × 0.059) eV.^70^ As per requirements, we can modulate the values of pH from 0 to 14. In the present work, we have taken pH = 0 (i.e., acidic environment).

Figure S14 (see in Supporting Information) shows the band edge position and redox potential at pH = 0 of the β-Sb monolayer using HSE, B3LYP, and GW functionals. Using the each method, the reduction and oxidation potential are situated in the band gap region, which means that the conduction band edge lies above the reduction potential of H^+^/H_2_ and also the valence band edge is lower than oxidation potential of O_2_/H_2_O. From this point of view, β-Sb and Bi@β-Sb monolayers can simultaneously produce both hydrogen and oxygen. It was also seen that the HER overpotential is relatively lower than the band edge position of CBM for reduction potential of H^+^/H_2_. It means that the Bi@β-Sb monolayer significantly creates the photogenerated electrons to drive the efficient HER mechanism using the results of HSE, B3LYP, and GW functionals for the Bi@β-Sb monolayer system. Also, the OER overpotential is lower than the energy differences between the band edge position of VBM and the oxidation potential of O_2_/H_2_O using B3LYP and GW functionals for the Bi@β-Sb monolayer system. It means that the Sb monolayer will be efficient for photogenerated holes to drive the OER mechanism. This indicates that β-Sb and Bi@β-Sb monolayers transfer the photoexcited holes and electrons to the water easily to produce both oxygen and hydrogen simultaneously at pH = 0. According to the abovementioned descriptions, the Bi@β-Sb monolayer is a superior candidate for photocatalytic performance for both hydrogen and oxygen production.

## Conclusions

We investigated a novel 2D β-Sb monolayer with a single Sb atom replacement with a Bi atom as an excellent candidate for photocatalysis. The 2D β-Sb monolayers are energetically, dynamically, and thermally stable, and it is confirmed on the basis of cohesive energy, phonon band structure, and AIMD simulations. Also, the 2D β-Sb monolayer with single Sb replaced by As, Bi, Sn, and Te atoms are energetically and thermally stable. The significantly larger excitonic binding energy is 0.604 eV, due to which it can effectively confine the electrons and holes. Therefore, the β-Sb monolayer will be very useful in the field of photocatalytic activity and optoelectronic devices. Furthermore, we found that the Bi@β-Sb monolayer shows a superior catalytic activity toward HER, OER, and ORR. The lower overpotential (η_OER_) for OER on the Bi@β-Sb monolayer is 0.25 V and ORR overpotential (η_ORR_) for the same configuration is 0.31 V. Also, the lowest energy barrier of the rate-determining step of HER activity on the Bi@β-Sb monolayer is 0.06 eV. Therefore, we expect the 2D Bi@β-Sb monolayer to be a highly efficient candidate for versatile HER/OER/ORR multifunctional photocatalysts. The finding results provide a useful direction to promote the advancement of cost-effective and high-performance photocatalysts toward renewable energy production.

## Supporting Information Available

Phonon band structure of pristine β-Sb; variation of total energy and geometry snapshot of the pristine β-Sb monolayer and As@β-Sb, Bi@β-Sb, Sn@β-Sb, and Te@β-Sb monolayers for 5 ps during MD simulation at 1000 K; 2D charge density profile and projected crystal orbital Hamilton population (−pCOHP) analysis of the pristine β-Sb monolayer and As@β-Sb, Bi@β-Sb, Sn@β-Sb, and Te@β-Sb monolayers; lowest energy configurations of the adsorption behavior of H atom on the surface of the β-Sb monolayer; charge density difference profile with top and side views for H atoms adsorbed on the surface of As@β-Sb, Bi@β-Sb, Sn@β-Sb, and Te@β-Sb monolayers; most favorable lowest energy configurations of O, OH, and OOH on the pristine β-Sb monolayer and As@β-Sb, Bi@β-Sb, Sn@β-Sb, and Te@β-Sb monolayers; −pCOHP analysis when H, O, OH, and OOH adsorb on the surface of the Bi@β-Sb monolayer, optical properties of the pristine β-Sb monolayer; decomposed p-orbital contribution in the electronic band structure of the pristine β-Sb monolayer; band alignment of the β-Sb and β-Sb@Bi monolayer; zero-point energy corrections and entropy (TS) contributions; and computational details about the HER/OER/ORR mechanism (PDF)
