# Supplementary material for: Theoretical Prediction of a Bi-Doped β-Antimonene Monolayer as a Highly Efficient Photocatalyst for Oxygen Reduction and Overall Water Splitting
Source: ACS Appl Mater Interfaces. 2021 Nov 16;13(47):56254–64. doi: 10.1021/acsami.1c18191 (PMC8640967; doi:10.1021/acsami.1c18191)
Supplement: Supplementary file 1 — am1c18191_si_001.pdf [file am1c18191_si_001.pdf]

Supporting Information

Theoretical Prediction of a Bi-Doped β-Antimonene Monolayer as a Highly Efficient Photocatalyst for Oxygen Reduction and Overall Water Splitting†

Deobrat Singh*[1] and Rajeev Ahuja* [1,2]

[1]*Condensed Matter Theory Group, Materials Theory Division, Department of Physics and Astronomy, Uppsala University, Box 516, 75120 Uppsala, Sweden*

[2]*Department of Physics, Indian Institute of Technology Ropar, Rupnagar 140001, Punjab, India*

(Dated: November 13, 2021)

*deobrat.singh@physics.uu.se

*rajeev.ahuja@physics.uu.se

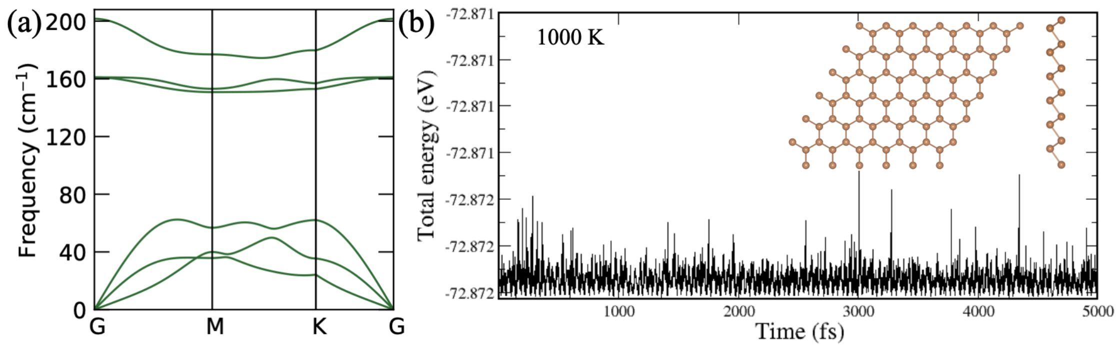

Figure S1. (a) Phonon dispersion spectra of pristine β-Sb monolayer. (b) The change of total energy at 1000 K as a function of time with a (6x6x1) supercell. The inserted displayed the relaxed β-sb monolayer after the AIMD simulation.

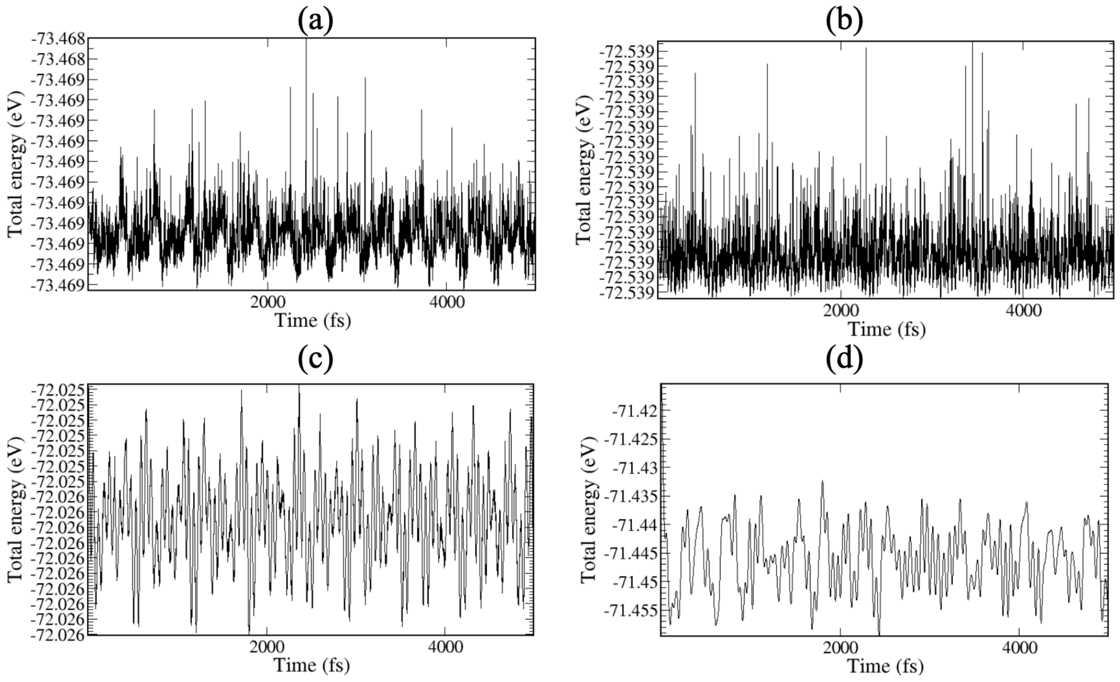

Figure S2. The change of total energy at 1000 K as a function of time with a (6x6x1) supercell. Single Sb atom replaced by As, Bi, Sn and Te named as (a) β-Sb@As, (b) β-Sb@Bi, (c) β-Sb@Sn and (d) β-Sb@Te monolayer.

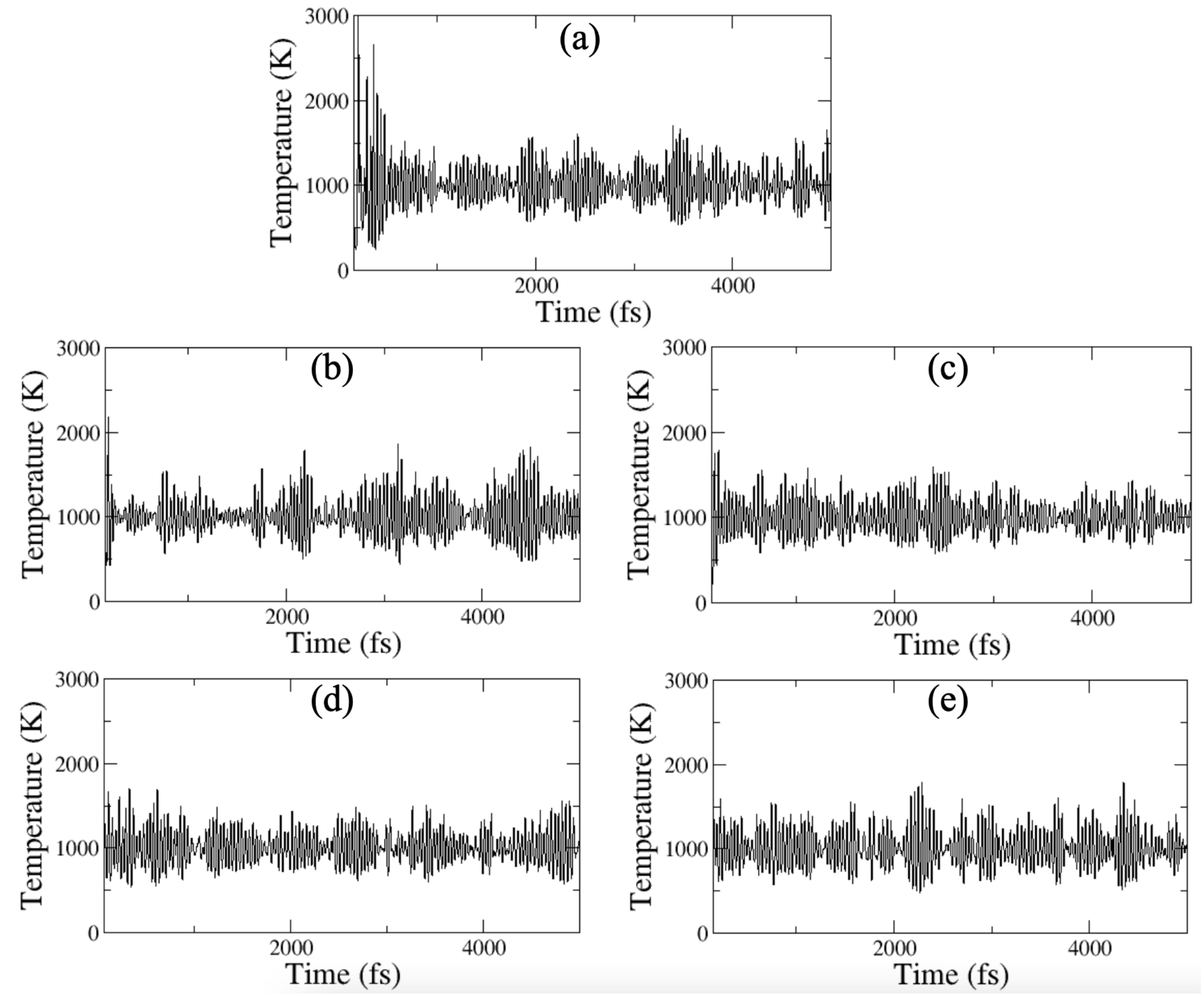

Figure S3. Fluctuations of temperature as a function of simulation time at 1000 K. (a) Pristine β-Sb monolayer and single Sb atom replaced by As, Bi, Sn and Te named as (b) β-Sb@As, (c) β-Sb@Bi, (d) β-Sb@Sn and (e) β-Sb@Te monolayer.

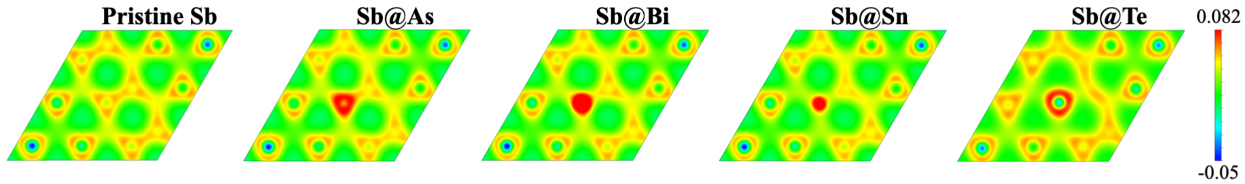

Figure S4. 2D charge density profile of the pristine β-Sb, single Sb atom replaced by As, Bi, Sn and Te named as β-Sb@As, β-Sb@Bi, β-Sb@Sn and β-Sb@Te system. The red color indicates the charge accumulation.

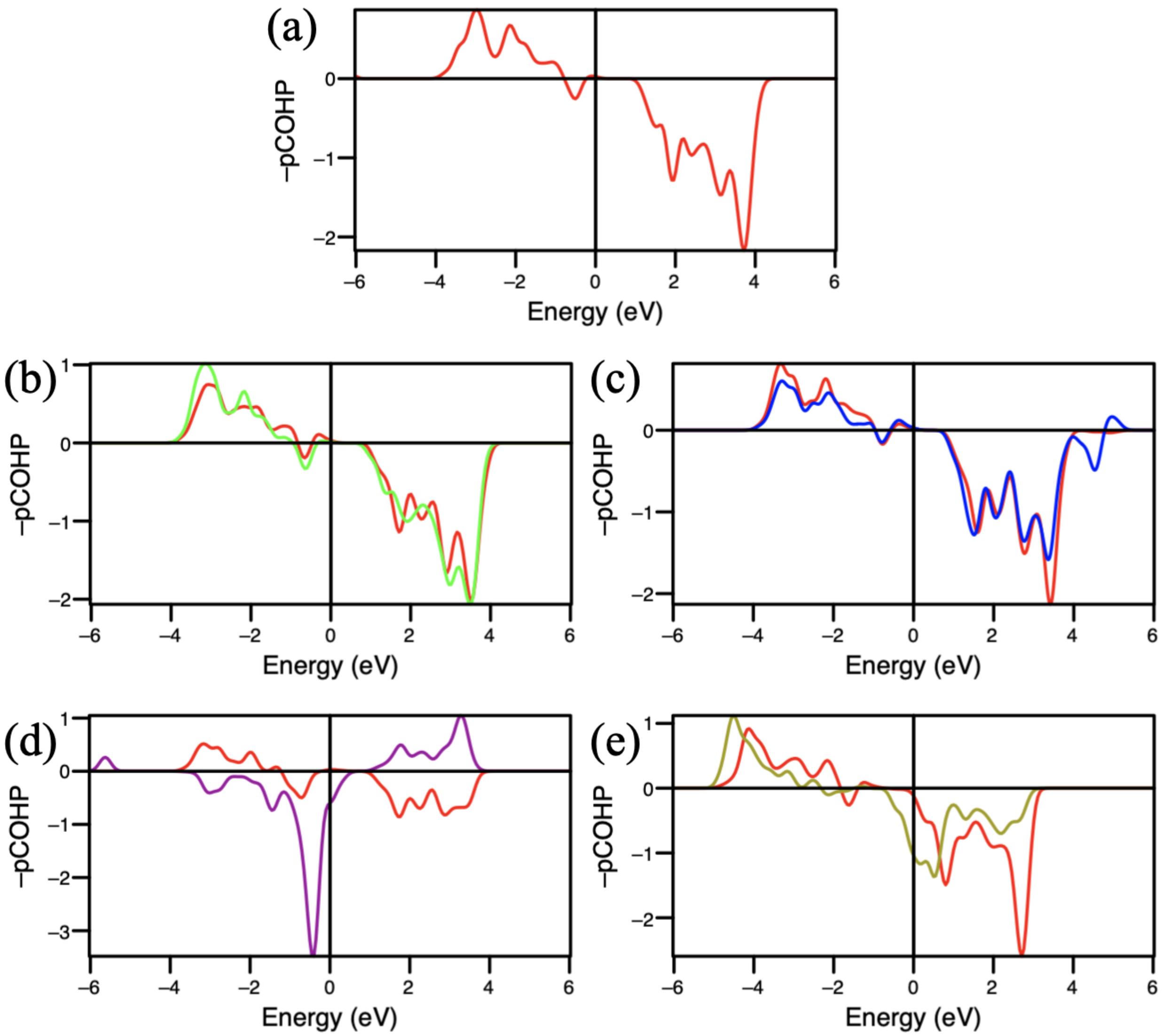

Figure S5. Projected crystal orbital Hamilton population (-pCOHP) analysis for (a) pristine β-Sb, (b) β-Sb@As (c) β-Sb@Bi (d) β-Sb@Sn and (e) β-Sb@Te of the pairs Sb-Sb, Sb-As, Sb-Bi, Sb-Sn and Sb-Te represented by red, green, blue, violet and olive-green, respectively. The zero vertical line shows the Fermi energy. The -pCOHP gives information about the contribution of a particular bond (bonding or anti-bonding) to the band energy. All the plots show bonding contribution in the up side (+y-axis) and the anti-bonding contribution is down side (-y-axis).

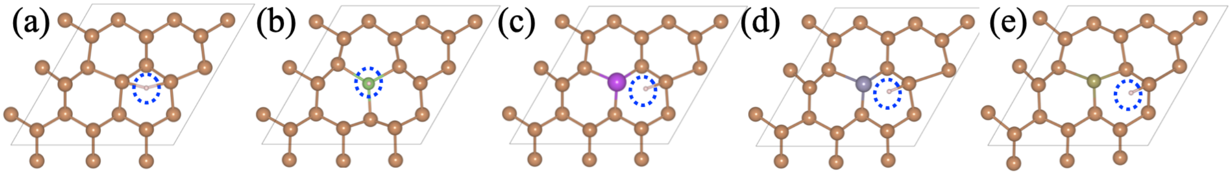

Figure S6. The lowest energy configurations of the adsorption behavior of H atom on the surface of (a) pristine β-Sb monolayer, (b) β-Sb@As, (c) β-Sb@Bi, (d) β-Sb@Sn and (e) β-Sb@Te systems. The dotted circle represents the adsorbed H atom.

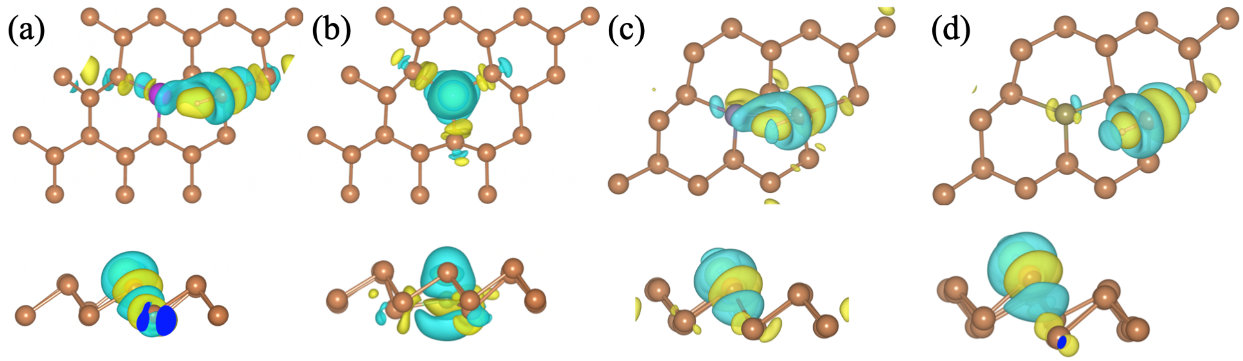

Figure S7. The charge density difference profile with top and side view for H-atom adsorbed on the surface of (a) β-Sb@As, (b) β-Sb@Bi, (c) β-Sb@Sn and (d) β-Sb@Te system. Yellow/blue color represents the electron accumulation/depletion.

Table S1. The zero-point energy (ZPE) corrections and entropy (TS) contributions to Gibbs free energies at 298.15 K.

Species	ZPE (eV)	TS (eV)
H_2	0.27	0.40
H_2O	0.57	0.67
O_2	0.11	0.63
O^*	0.05	0.00
HO^*	0.34	0.06
HOO^*	0.42	0.00

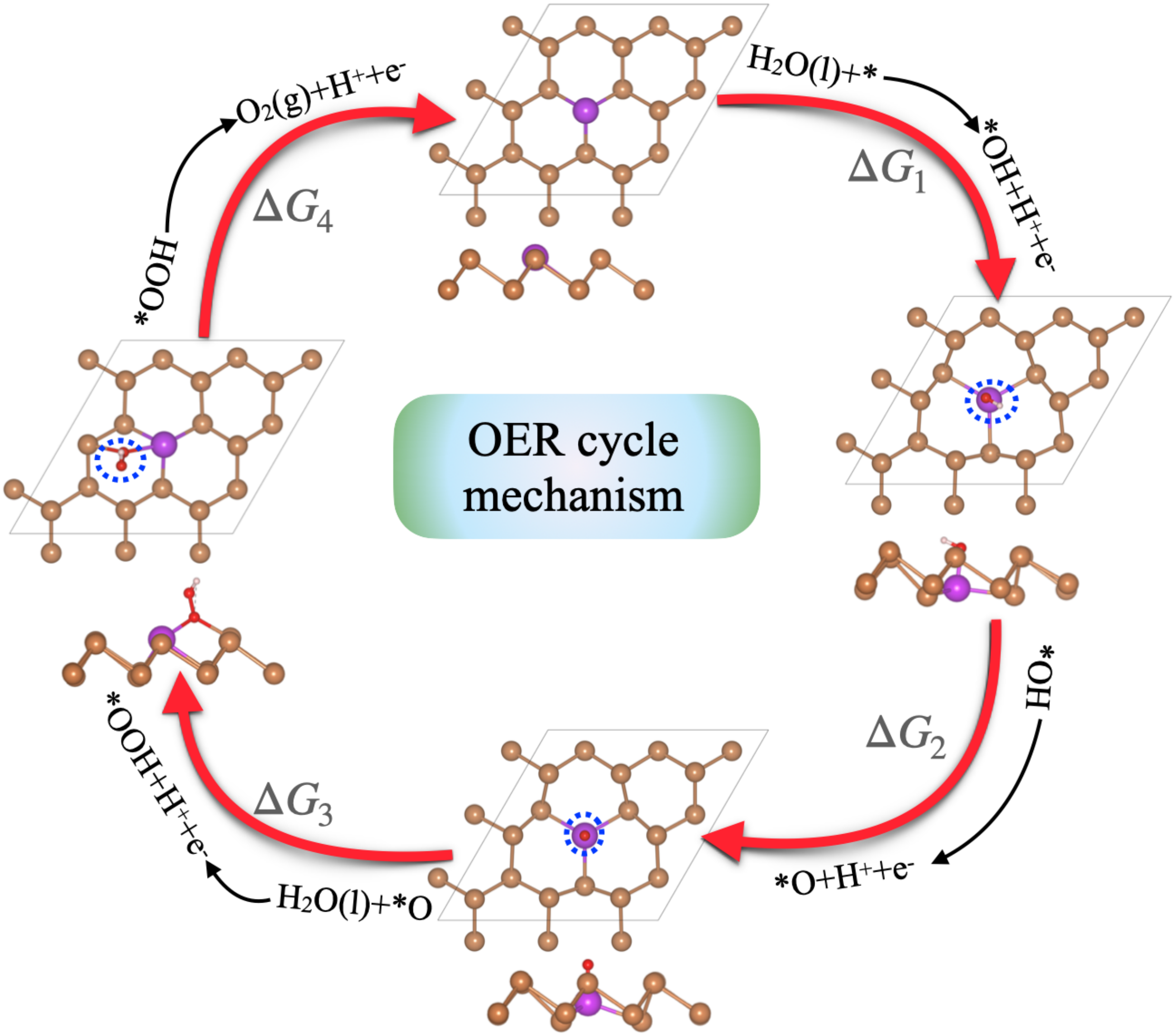

Figure S8. The $4e^{-}$ OER reaction cycle by the single active site mechanism with the lowest energy configuration for intermediates.

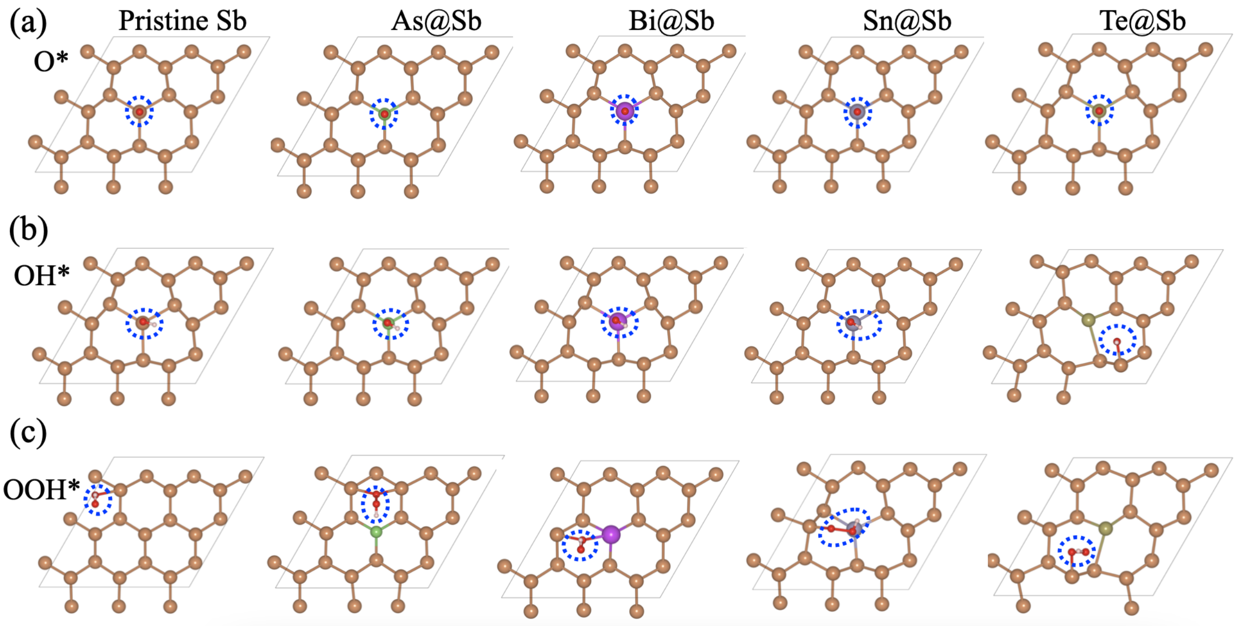

Figure S9. The most favourable lowest energy configurations of O, OH and OOH adsorb on pristine β-Sb monolayer, β-Sb@As, β-Sb@Bi, β-Sb@Sn and β-Sb@Te systems. The most stable adsorption configurations of (a) O, (b) OH and (c) OOH on β-Sb monolayer, β-Sb@As, β-Sb@Bi, β-Sb@Sn and β-Sb@Te systems.

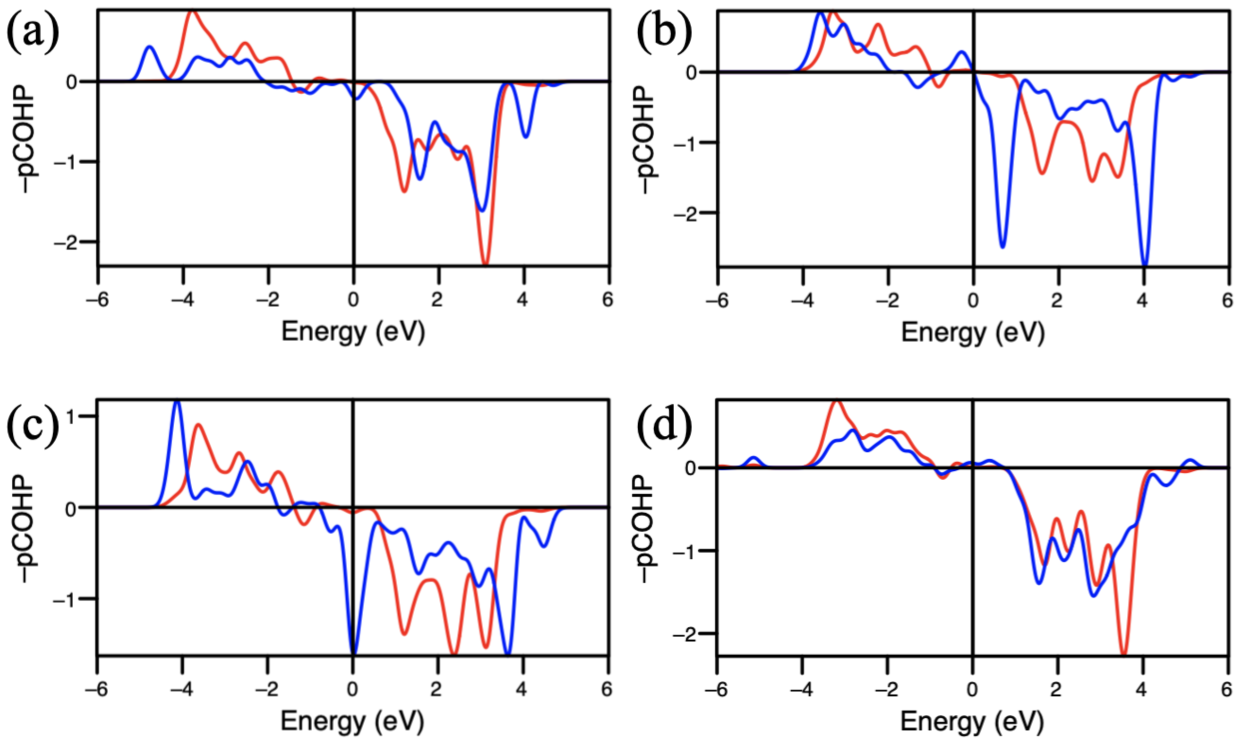

Figure S10. Projected crystal orbital Hamilton population (-pCOHP) analysis when (a) H, (b) O, (c) OH and (d) OOH adsorb on the high-performance catalytic surface of β-Sb@Bi for the pairs Sb-Sb and Sb-Bi represented by red and blue color respectively. The zero vertical line shows the Fermi energy. The -pCOHP gives information about the contribution of a particular bond (bonding or anti-bonding) to the band energy. All the plots show bonding contribution in the up side (+y-axis) and the anti-bonding contribution is down side (-y-axis).

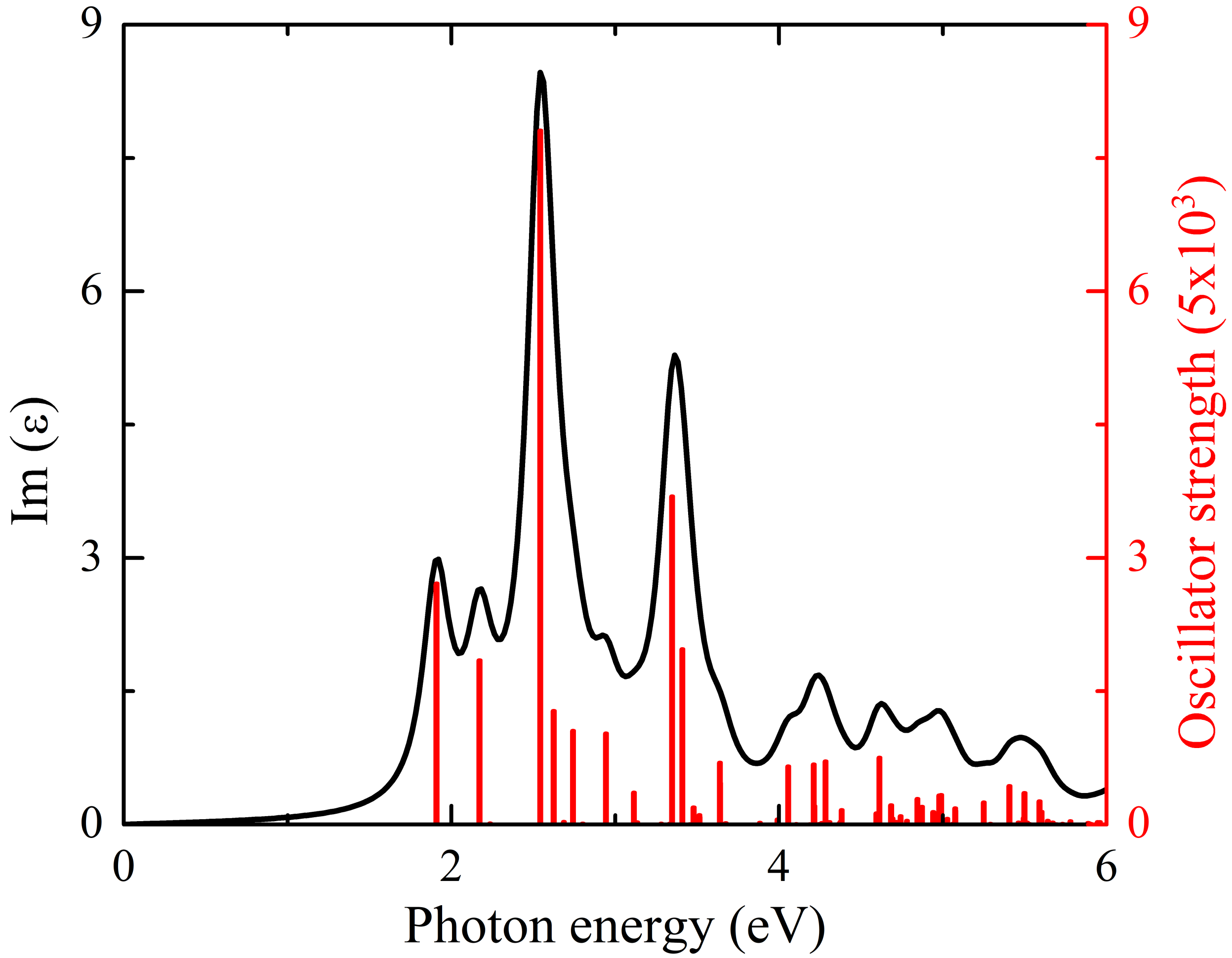

Figure S11. Imaginary part of dielectric function and corresponding oscillator strength of β-Sb monolayer.

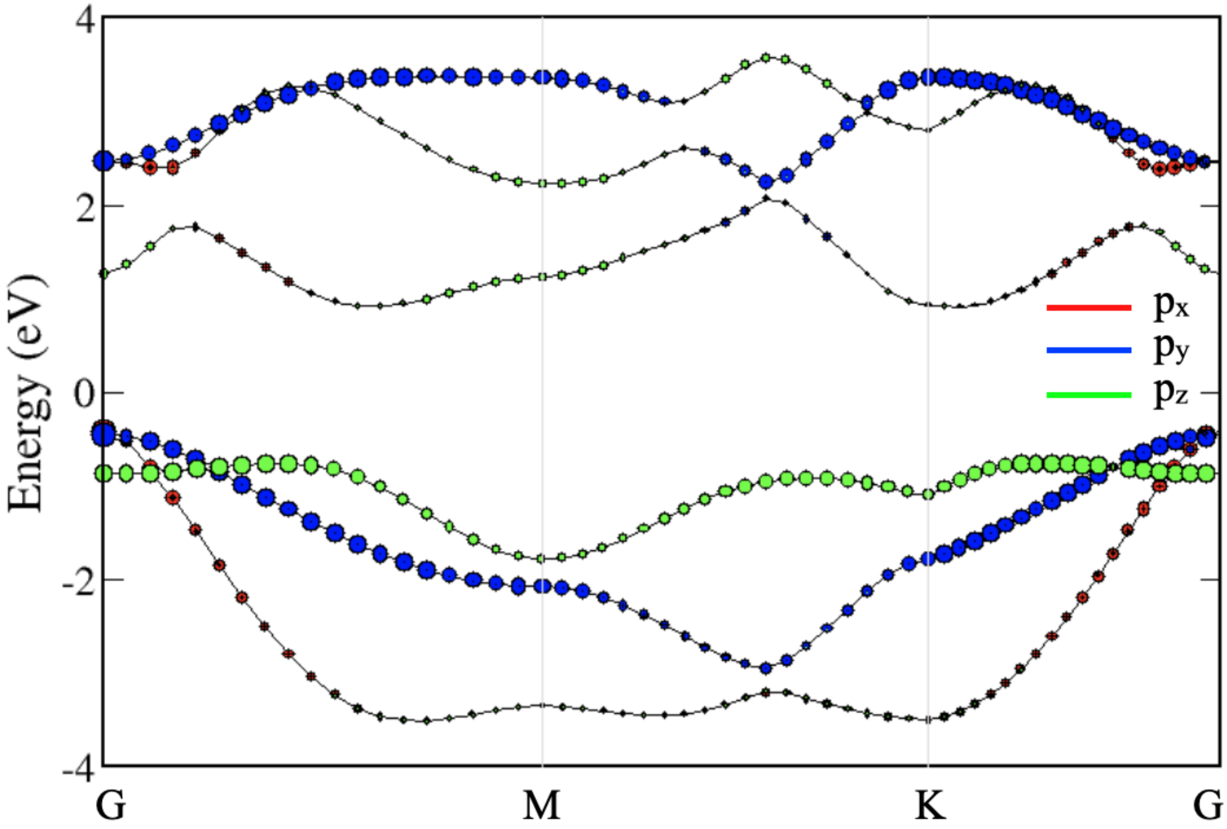

Figure S12. Decomposed p-orbital contribution in the electronic band structure of β-Sb monolayer.

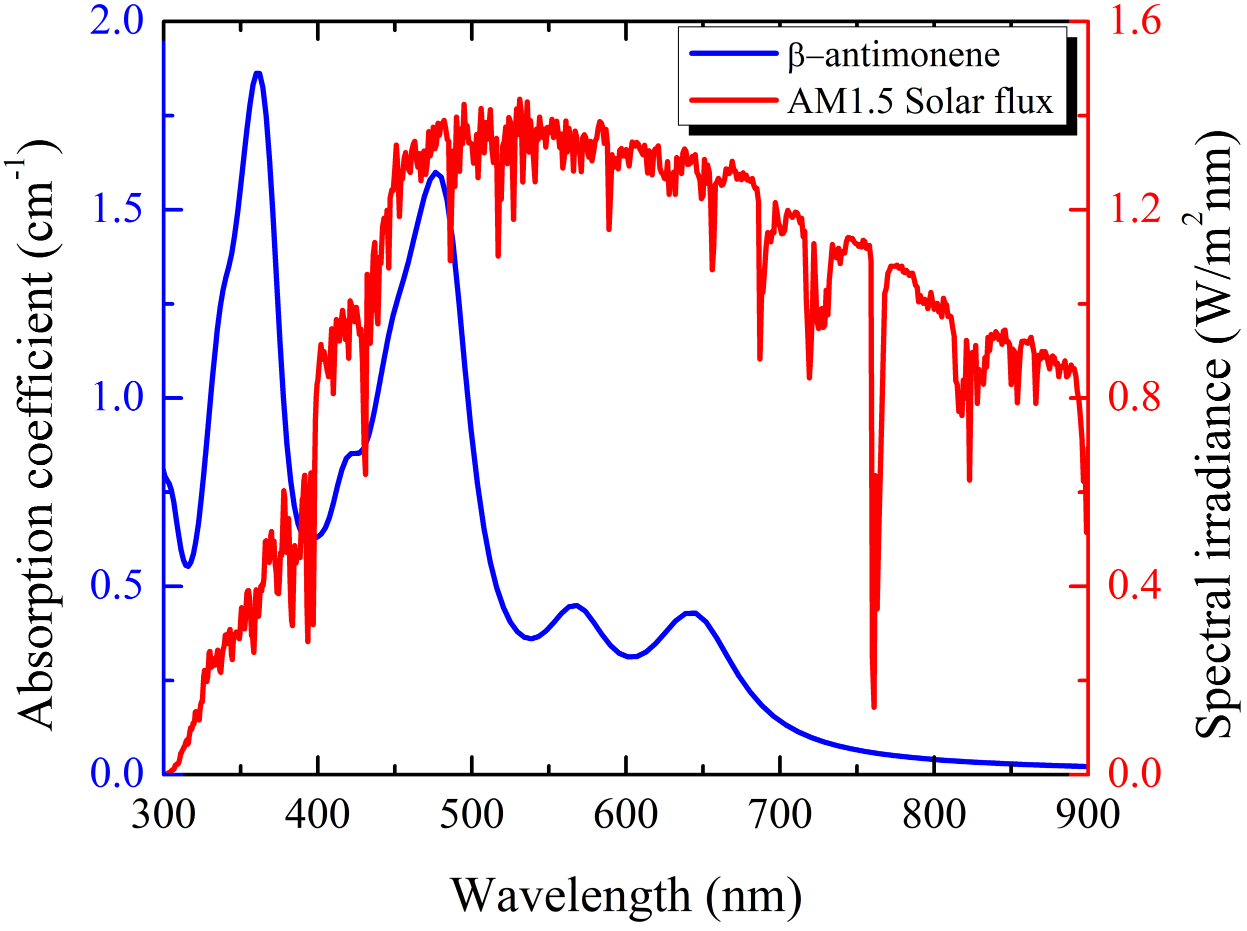

Figure S13. Calculated light absorption spectrum as a function of photon energy of β-Sb monolayer using GW plus BSE method superimposed to the incident AM1.5G solar flux.

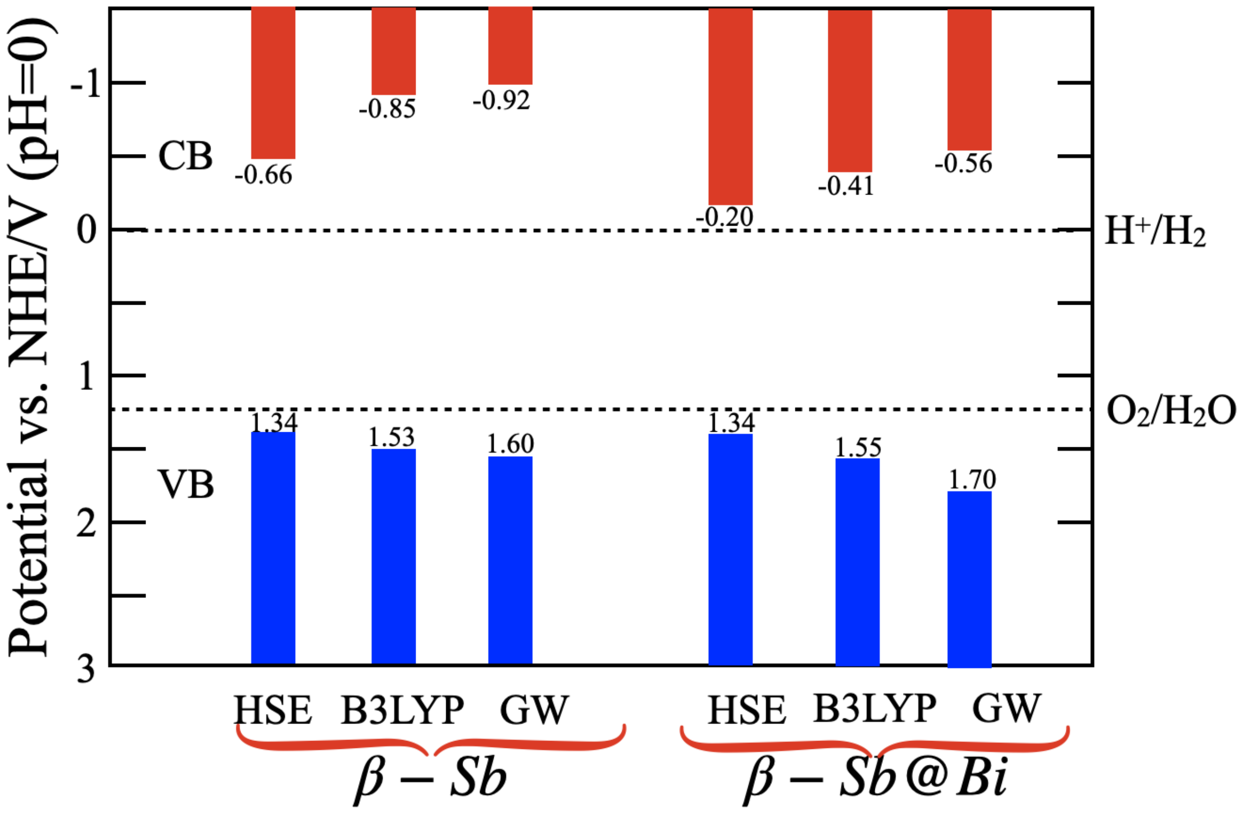

Figure S14. Band alignment of β-Sb and β-Sb@Bi monolayer.

I. COMPUTATIONAL DETAILS ABOUT HER, OER AND ORR

The reactions mechanism of HER, OER and ORR activities are investigated by calculating the reaction free energy of each step.

A. ΔG for HER

The standard hydrogen electrode (U_{SHE}) was theoretically defined in solution [pH = 0, p(H_2) = 1 bar]. The overall HER pathway can be described by,

$$H^+(aq.) + e^- +^* \rightarrow \frac{1}{2}H_2{}^* \rightarrow \frac{1}{2}H_2(g) +^* \ \ (\Delta G = 0\ eV) \tag{1}$$

where $H^+(aq.) + e^-$ is an initial state, H^* an intermediate adsorbed, and $H_2(g)$ is the final product. Here, "$*$" represents the lowest adsorption site for intermediates, (aq.) and (g) represents the aqueous solution and gas phases, respectively. The Gibbs free energy of the adsorption of intermediate hydrogen (ΔG_H*)on the catalyst is a key descriptor for the HER mechanism of the catalyst [1] and is defined by,

$$\Delta G_H* = \Delta E_H* + \Delta E_{ZPE} - T\Delta S_H* \tag{2}$$

in which ΔE_H is the hydrogen adsorption energy and defined as,

$$\Delta E_H* = E_{nH}* - E_{(n-1)H}* - \frac{1}{2}E_{H_2} \tag{3}$$

where $E_{nH}*$, $E_{(n-1)H}*$ shows the investigated energy of the catalysts with nH and (n-1)H adsorption. ΔE_{ZPE} and $T\Delta S_H*$ are the difference of zero-point energy and difference in entropy between adsorbed and gas phases, respectively. Contributions from catalysts both ΔE_{ZPE} and $T\Delta S_H*$ are small and neglected, and the vibration frequencies of the H^* adsorbed on the catalysts are not sensitive to coverage [2].

Therefore, ΔE_{ZPE} is described by

$$\Delta E_{ZPE} = E_{ZPE}^{H^*} - \frac{1}{2}E_{ZPE}^{H_2^*} \tag{4}$$

And the value of ΔS_H is defined as,

$$\Delta S_H \cong -\frac{1}{2}S^0_{H_2} \tag{5}$$

where $\frac{1}{2}S^0_{H_2}$ is the entropy of H_2 under the standard condition and the value is 130 $mol^{-1}K^{-1}$ [3].

The ideal value for HER is $\Delta G_H* = 0$. Also, the smaller $\mid \Delta G_H* \mid$, the better HER performance will be.

The theoretical overpotential η_{HER} for HER [5] which is determined by ΔG_H*,

$$\eta_{HER} = -\frac{\mid \Delta G_{H}* \mid}{e} \tag{6}$$

In the acidic environment, the overall OER could be described in Eqn. (6), which happens on the cathode of water splitting and the metal air battery during charge [4],

$$H_2O \rightarrow O_2 + 4H^* + 4e^- \tag{7}$$

The OER mechanism proceeds through a 4e$^-$ transferred reaction pathway as follows,

$$H_2O(l) +^* \rightarrow OH^* + H^+ + e^- \tag{8}$$

$$OH^* \rightarrow O^* + H^+ + e^- \tag{9}$$

$$H_2O(l) + O^* \rightarrow OOH^* + H^+ + e^- \tag{10}$$

$$OOH^* \rightarrow O_2(g) + H^+ + e^- \tag{11}$$

where * refers to the active site on the catalysts. (g) and (l) represents the gas and liquid phase of oxygen and water molecules, respectively.

The ORR reaction mechanism occurs via elementary steps takes the reverse direction of OER mechanism [6],

$$O_2 + 4H^* + 4e^- \rightarrow H_2O \tag{12}$$

In the acidic solution, the ORR proceeds via the 4e$^-$-transfer pathway can be written as below,

$$^* + O_2(g) + H^+ + e^- \rightarrow OOH^* \tag{13}$$

$$OOH^* + H^+ + e^- \rightarrow H_2O(l) + O^* \tag{14}$$

$$O^* + H^+ + e^- \rightarrow OH^* \tag{15}$$

$$OH^* + H^+ + e^- \rightarrow H_2O(l) +^* \tag{16}$$

The theoretically Gibbs free energy difference ($\Delta G_{OH}*$, $\Delta G_{O}*$, and $\Delta G_{OOH}*$) of each step involving one e^- transfer is defined by the following equation,

$$\Delta G = \Delta E + \Delta ZPE - T\Delta S + \Delta G_U + \Delta G_{pH} \tag{17}$$

Here, ΔE, ΔZPE, and ΔS displayed the energy difference of adsorption, zero-point energy and entropy between adsorbed state and freestanding state, respectively. The values of ΔE was calculated by DFT, and the values for ΔZPE, and ΔS were investigated through DFT calculations and the standard thermodynamic data, as presented in Table S1. ΔG_U=-eU, where e is the elementary charge, U is the applied electrode potential. And the last term ΔG_{pH}=-$k_B T$ $ln[H^+]$=pH×$k_B T$ $ln10$ originating from the effect of pH value of the electrolyte. The free energy changes during 4e^- pathways for OER could be expressed as; ΔG_1=$\Delta G_{OH}*$, ΔG_2=ΔG_O*-$\Delta G_{OH}*$, ΔG_3=$\Delta G_{OOH}*$-ΔG_O*, and ΔG_4=4.92-$\Delta G_{OOH}*$. And for ORR steps; ΔG_a=$\Delta G_{OOH}*$-4.92, ΔG_b=ΔG_O*-$\Delta G_{OOH}*$, ΔG_c=$\Delta G_{OH}*$-ΔG_O*, and ΔG_d=$\Delta G_{OH}*$.

The overpotential ($\eta_{OER/ORR}$) that could be used to evaluate the catalytic performance of REOs and ORR obtained from the following equations;

$$\eta_{OER} = max\{\frac{\Delta G_1, \Delta G_2, \Delta G_3, \Delta G_4}{e}\} - 1.23 \quad (18)$$

$$\eta_{ORR} = max\{\frac{-\Delta G_a, -\Delta G_b, -\Delta G_c, -\Delta G_d}{e}\} + 1.23 \quad (19)$$

REFERENCES

[1] Tsai, C.; Abild-Pedersen, F.; Nørskov, J. K. Tuning the MoS_2 Edge-site Activity for Hydrogen Evolution via Support Interactions. Nano letters **2014**, 14(3), 1381–1387.

[2] Gao, G.; O'Mullane, A. P.; Du, A. 2D MXenes: A New Family of Promising Catalysts for the Hydrogen Evolution Reaction. ACS Catalysis **2017**, 7(1), 494–500.

[3] Atkins, P.; De Paula, J.; Friedman, R. Physical Chemistry: Quanta, Matter, and Change. Oxford University Press, USA **2014**.

[4] Rossmeisl, J.; Qu, Z. W.; Zhu, H.; Kroes, G. J.; Nørskov, J. K. Electrolysis of Water on Oxide Surfaces. Journal of Electroanalytical Chemistry **2007**, 607(1–2), 83–89.

[5] Wang, J.; Fan, Y.; Qi, S.; Li, W.; Zhao, M. Bifunctional HER/OER or OER/ORR Catalytic Activity of Two-Dimensional $TM_3(HITP)_2$ with TM= Fe–Zn. The Journal of Physical Chemistry C **2020**, 124(17), 9350–9359.

[6] Nørskov, J. K.; and Rossmeisl, J.; Logadottir, A.; Lindqvist, L. R. K. J.; Kitchin, J. R.; Bligaard, T.; Jonsson, H. Origin of the Overpotential for Oxygen Reduction at a Fuel-cell Cathode. The Journal of Physical Chemistry B **2004**, 108(46), 17886–17892.
